# In-Depth Review of Augmented Reality: Tracking Technologies, Development Tools, AR Displays, Collaborative AR, and Security Concerns

**DOI:** 10.3390/s23010146

**Published:** 2022-12-23

**Authors:** Toqeer Ali Syed, Muhammad Shoaib Siddiqui, Hurria Binte Abdullah, Salman Jan, Abdallah Namoun, Ali Alzahrani, Adnan Nadeem, Ahmad B. Alkhodre

**Affiliations:** 1 Faculty of Computer and Information Systems, Islamic University of Madinah, Medina 42351, Saudi Arabia; 2 School of Social Sciences and Humanities, National University of Science and Technology (NUST), Islamabad 44000, Pakistan; 3 Malaysian Institute of Information Technology, Universiti Kuala Lumpur, Kuala Lumpur 50250, Malaysia; 4 Department of Computer Science, Bacha Khan University Charsadda, Charsadda 24420, Pakistan

**Keywords:** trusted augmented reality, augmented reality review, collaborative augmented reality, virtual reality review, display and tracking technology, display technologies in augmented reality

## Abstract

Augmented reality (AR) has gained enormous popularity and acceptance in the past few years. AR is indeed a combination of different immersive experiences and solutions that serve as integrated components to assemble and accelerate the augmented reality phenomena as a workable and marvelous adaptive solution for many realms. These solutions of AR include tracking as a means for keeping track of the point of reference to make virtual objects visible in a real scene. Similarly, display technologies combine the virtual and real world with the user’s eye. Authoring tools provide platforms to develop AR applications by providing access to low-level libraries. The libraries can thereafter interact with the hardware of tracking sensors, cameras, and other technologies. In addition to this, advances in distributed computing and collaborative augmented reality also need stable solutions. The various participants can collaborate in an AR setting. The authors of this research have explored many solutions in this regard and present a comprehensive review to aid in doing research and improving different business transformations. However, during the course of this study, we identified that there is a lack of security solutions in various areas of collaborative AR (CAR), specifically in the area of distributed trust management in CAR. This research study also proposed a trusted CAR architecture with a use-case of tourism that can be used as a model for researchers with an interest in making secure AR-based remote communication sessions.

## 1. Introduction

Augmented reality (AR) is one of the leading expanding immersive experiences of the 21st century. AR has brought a revolution in different realms including health and medicine, teaching and learning, tourism, designing, manufacturing, and other similar industries whose acceptance accelerated the growth of AR in an unprecedented manner [1,2,3]. According to a recent report in September 2022, the market size of AR and VR reached USD 27.6 billion in 2021, which is indeed estimated to reach USD 856.2 billion by the end of the year 2031 [4]. Big companies largely use AR-based technologies. For instance, Amazon, one of the leading online shopping websites, uses this technology to make it easier for customers to decide the type of furniture they want to buy. The rise in mobile phone technology also acted as an accelerator in popularizing AR. Earlier, mobile phones were not advanced and capable enough to run these applications due to their low graphics. Nowadays, however, smart devices are capable enough to easily run AR-based applications. A lot of research has been done on mobile-based AR. Lee et al. [5] developed a user-based design interface for educational purpose in mobile AR. To evaluate its conduct, fourth-grade elementary students were selected.

The adoption of AR in its various perspectives is backed up by a prolonged history. This paper presents an overview of the different integrated essential components that contribute to the working framework of AR, and the latest developments on these components are collected, analyzed, and presented, while the developments in the smart devices and the overall experience of the users have changed drastically [6]. The tracking technologies [7] are the building blocks of AR and establish a point of reference for movement and for creating an environment where the virtual and real objects are presented together. To achieve a real experience with augmented objects, several tracking technologies are presented which include techniques such as sensor-based [8], markerless, marker-based [9,10], and hybrid tracking technologies. Among these different technologies, hybrid tracking technologies are the most adaptive. As part of the framework constructed in this study, the simultaneous localization and mapping (SLAM) and inertial tracking technologies are combined. The SLAM technology collects points through cameras in real scenes while the point of reference is created using inertial tracking. The virtual objects are inserted on the relevant points of reference to create an augmented reality. Moreover, this paper analyzes and presents a detailed discussion on different tracking technologies according to their use in different realms i.e., in education, industries, and medical fields. Magnetic tracking is widely used in AR systems in medical, maintenance, and manufacturing. Moreover, vision-based tracking is mostly used in mobile phones and tablets because they have screen and camera, which makes them the best platform for AR. In addition, GPS tracking is useful in the fields of military, gaming, and tourism. These tracking technologies along with others are explained in detail in Section 3.

Once the points of reference are collected after tracking, then another important factor that requires significant accuracy is to determine at which particular point the virtual objects have to be mixed with the real environment. Here comes the role of display technologies that gives the users of augmented reality an environment where the real and virtual objects are displayed visually. Therefore, display technologies are one of the key components of AR. This research identifies state-of-the-art display technologies that help to provide a quality view of real and virtual objects. Augmented reality displays can be divided into various categories. All have the same task to show the merged image of real and virtual content to the user’s eye. The authors have categorized the latest technologies of optical display after the advancements in holographic optical elements (HOEs). There are other categories of AR displays, such as video-based, eye multiplexed, and projected onto a physical surface. Optical see-through has two sub-categories, one is a free-space combiner and the other is a wave-guide combiner [11,12]. The thorough details of display technologies are presented in Section 4.

**Figure 1:**
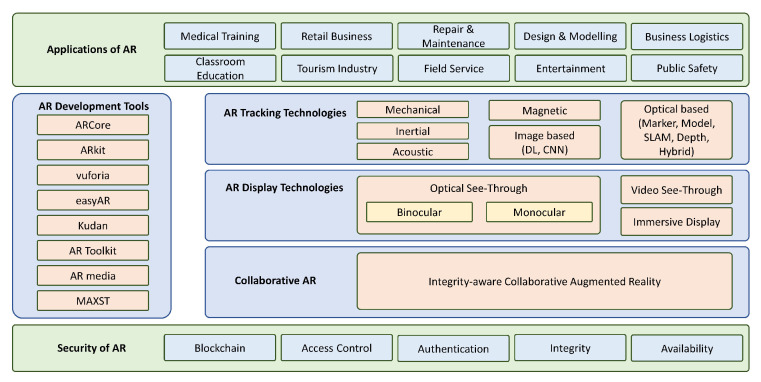
Overview of AR, VR, and collaborative AR applications, tools, and technologies.

To develop these AR applications, different tools are used depending on the type of application used. For example, to develop a mobile-based AR application for Android or iOS, ARToolKit [13] is used. However, FLARToolKit [14] is used to create a web-based application using Flash. Moreover, there are various plug-ins available that can be integrated with Unity [15] to create AR applications. These development tools are reviewed in Section 6 of this paper. Figure 1 provides an overview of reviewed topics of augmented reality in this paper.

After going through a critical review process of collaborative augmented reality, the research has identified that some security flaws and missing trust parameters need to be addressed to ensure a pristine environment is provided to the users. Hackers and intruders are always active to exploit different vulnerabilities in the systems and software, but the previous research conducted on collaborative augmented reality did not depict reasonable efforts made in this direction to make secure collaboration. To address the security flaws and to provide secure communication in collaborative augmented reality, this research considered it appropriate to come up with a security solution and framework that can limit danger and risks that may be posed in the form of internal and external attacks. To actualize the secure platform, this study came up with an architecture for presenting a secure collaborative AR in the tourism sector in Saudi Arabia as a case study. The focus of the case study is to provide an application that can guide tourists during their visit to any of the famous landmarks in the country. This study proposed a secure and trustful mobile application based on collaborative AR for tourists. In this application, the necessary information is rendered on screen and the user can hire a guide to provide more information in detail. A single guide can provide the services to a group of tourists visiting the same landmark. A blockchain network was used to secure the applications and protect the private data of the users [16,17]. For this purpose, we performed a thorough literature review for an optimized solution regarding security and tracking for which we studies the existing tracking technologies and listed them in this paper along with their limitations. In our use case, we used a GPS tracking system to track the user’s movement and provide the necessary information about the visited landmark through the mobile application.

**Figure 2:**
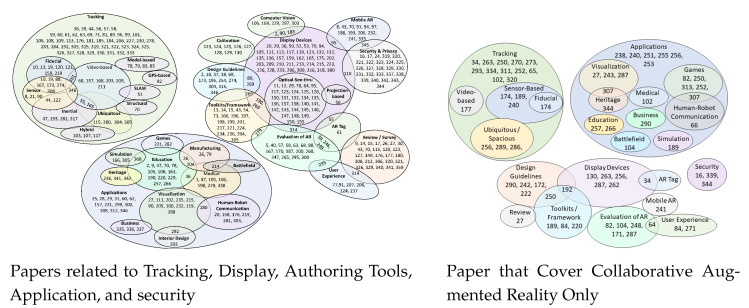
Classification of reviewed papers with respect to tracking, display, authoring tools, application, Collaborative and security.

Observing the fact that AR operates in an integrated fashion that combines different technologies including tracking technologies, display technologies, AR tools, collaborative AR, and applications of AR has encouraged us to explore and present these conceptions and technologies in detail. To facilitate researchers on these different techniques, the authors have explored the research previously conducted and presented it in a Venn diagram, as shown in Figure 2. Interested investigators can choose their required area of research in AR. As can be seen in the diagram, most research has been done in the area of tracking technologies. This is further divided into different types of tracking solutions including fiducial tracking, video-based tracking, and inertial tracking. Some papers lie in several categories for, example some papers such as [18,19,20] fall in both the fiducial tracking and sensor categories. Similarly, computer vision and display devices have some common papers, and inertial tracking and video-based tracking also have some papers in common. In addition, display devices share common papers with computer vision, mobile AR, design guidelines, tool-kits, evaluation, AR tags, and security and privacy of AR. Furthermore, visualization has different papers in common with business, interior design, and human–robot communication. While education shares some paper with gaming, simulation, medicine, heritage, and manufacturing. In short, we have tried to summarize all papers and further elaborate in their sections for the convenience of the reader.

Contribution: This research presents a comprehensive review of AR and its associated technologies. A review of state-of-the-art tracking and display technologies is presented followed by different essential components and tools that can be used to effectively create AR experiences. The study also presents the newly emerging technologies such as collaborative augmented reality and how different application interactions are carried out. During the review phase, the research identified that the AR-based solutions and particularly collaborative augmented reality solutions are vulnerable to external intrusion. It is identified that these solutions lack security and the interaction could be hijacked, manipulated, and sometimes exposed to potential threats. To address these concerns, this research felt the need to ensure that the communication has integrity; henceforth, the research utilizes the state-of-the-art blockchain infrastructure for the collaborating applications in AR. The paper further proposes complete secure framework wherein different applications working remotely have a real feeling of trust with each other [21].

**Outline**: This paper presents the overview of augmented reality and its applications in various realms in Section 2. In Section 3, tracking technologies are presented, while a detailed overview of the display technologies is provided in Section 4. Section 6 apprises readers on AR development tools. Section 7 highlights the collaborative research on augmented reality, while Section 8 interprets the AR interaction and input technologies. The paper presents the details of design guidelines and interface patterns in Section 9, while Section 10 discusses the security and trust issues in collaborative AR. Section 12 highlights future directions for research, while Section 13 concludes this research.

## 2. Augmented Reality Overview

**Figure 3:**
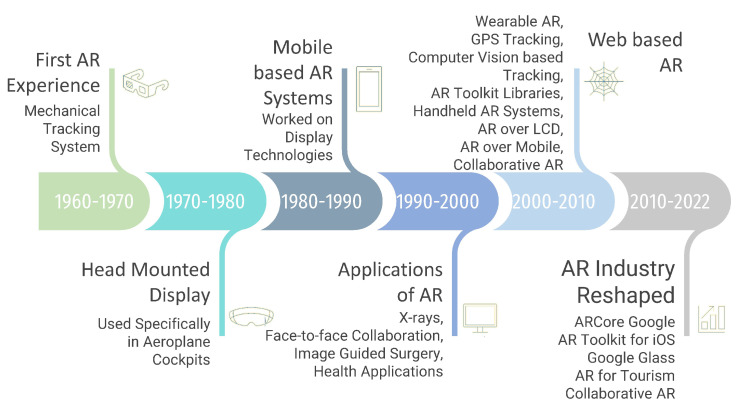
Augmented reality advancement over time for the last 60 years.

People, for many years, have been using lenses, light sources, and mirrors to create illusions and virtual images in the real world [22,23,24]. However, Ivan Sutherland was the first person to truly generate the AR experience. Sketchpad, developed at MIT in 1963 by Ivan Sutherland, is the world’s first interactive graphic application [25]. In Figure 3, we have given an overview of the development of AR technology from the beginning to 2022. Bottani et al. [26] reviews the AR literature published during the time period of 2006–2017. Moreover, Sereno et al. [27] use a systematic survey approach to detail the existing literature available on the intersection of computer-supported collaborative work and AR.

### 2.1. Head-Mounted Display

Ens et al. [28] review the existing work on design exploration for mixed-scale gestures where the Hololens AR display is used to interweave larger gestures with micro-gestures.

### 2.2. AR Towards Applications

ARToolKit tracking library [13] aimed to provide the computer vision tracking of a square marker in real-time which fixed two major problems, i.e., enabling interaction with real-world objects and secondly, the user’s viewpoint tracking system. Researchers conducted studies to develop handheld AR systems. Hettig et al. [29] present a system called “Augmented Visualization Box” to asses surgical augmented reality visualizations in a virtual environment. Goh et al. [30] present details of the critical analysis of 3D interaction techniques in mobile AR. Kollatsch et al. [31] introduce a system that creates and introduces the production data and maintenance documentation into the AR maintenance apps for machine tools which aims to reduce the overall cost of necessary expertise and the planning process of AR technology. Bhattacharyya et al. [32] introduce a two-player mobile AR game known as Brick, where users can engage in synchronous collaboration while inhabiting the real-time and shared augmented environment. Kim et al. [33] suggest that this decade is marked by a tremendous technological boom particularly in rendering and evaluation research while display and calibration research has declined. Liu et al. [34] expand the information feedback channel from industrial robots to a human workforce for human–robot collaboration development.

### 2.3. Augmented Reality for the Web

Cortes et al. [35] introduce the new techniques of collaboratively authoring surfaces on the web using mobile AR. Qiao et al. [36] review the current implementations of mobile AR, enabling technologies of AR, state-of-art technology, approaches for potential web AR provisioning, and challenges that AR faces in a web-based system.

### 2.4. AR Application Development

The AR industry was tremendously increasing in 2015, extending from smartphones to websites with head-worn display systems such as Google Glass. In this regard, Agati et al. [18] propose design guidelines for the development of an AR manual assembly system which includes ergonomics, usability, corporate-related, and cognition.

AR for Tourism and Education: Shukri et al. [37] aim to introduce the design guidelines of mobile AR for tourism by proposing 11 principles for developing efficient AR design for tourism which reduces cognitive overload, provides learning ability, and helps explore the content while traveling in Malaysia. In addition to it, Fallahkhair et al. [38] introduce new guidelines to make AR technologies with enhanced user satisfaction, efficiency, and effectiveness in cultural and contextual learning using mobiles, thereby enhancing the tourism experience. Akccayir et al. [39] show that AR has the advantage of placing the virtual image on a real object in real time while pedagogical and technical issues should be addressed to make the technology more reliable. Salvia et al. [40] suggest that AR has a positive impact on learning but requires some advancements.

Sarkar et al. [41] present an AR app known as ScholAR. It introduces enhancing the learning skills of the students to inculcate conceptualizing and logical thinking among sevemth-grade students. Soleiman et al. [42] suggest that the use of AR improves abstract writing as compared to VR.

### 2.5. AR Security and Privacy

Hadar et al. [43] scrutinize security at all steps of AR application development and identify the need for new strategies for information security, privacy, and security, with a main goal to design and introduce capturing and mapping concerns. Moreover, in the industrial arena, Mukhametshin et al. [44] focus on developing sensor tag detection, tracking, and recognition for designing an AR client-side app for Siemen Company to monitor the equipment for remote facilities.

## 3. Tracking Technology of AR

Tracking technologies introduce the sensation of motion in the virtual and augmented reality world and perform a variety of tasks. Once a tracking system is rightly chosen and correctly installed, it allows a person to move within a virtual and augmented environment. It further allows us to interact with people and objects within augmented environments. The selection of tracking technology depends on the sort of environment, the sort of data, and the availability of required budgets. For AR technology to meet Azuma’s definition of an augmented reality system, it must adhere to three main components:

1. it combines virtual and the real content;
2. it is interactive in real time;
3. is is registered in three dimensions.

The third condition of being “registered in three dimensions” alludes to the capability of an AR system to project the virtual content on physical surroundings in such a way that it seems to be part of the real world. The position and orientation (pose) of the viewer concerning some anchor in the real world must be identified and determined for registering the virtual content in the real environment. This anchor of the real world may be the dead-reckoning from inertial tracking, a defined location in space determined using GPS, or a physical object such as a paper image marker or magnetic tracker source. In short, the real-world anchor depends upon the applications and the technologies used. With respect to the type of technology used, there are two ways of registering the AR system in 3D:

- Determination of the position and orientation of the viewer relative to the real-world anchor: registration phase;
- Upgrading of viewer’s pose with respect to previously known pose: tracking phase.

**Figure 4:**
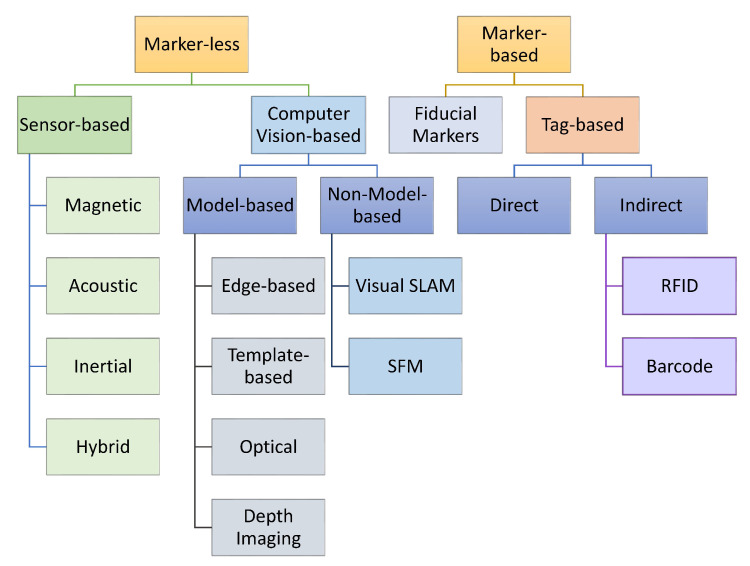
Categorization of augmented reality tracking techniques.

In this document, the word “tracking” would define both phases as common terminology. There are two main types of tracking techniques which are explained as follows (depicted in Figure 4).

### 3.1. Markerless Tracking Techniques

Markerless tracking techniques further have two types, one is sensor based and another is vision based.

#### 3.1.1. Sensor-Based Tracking

**Figure 5:**
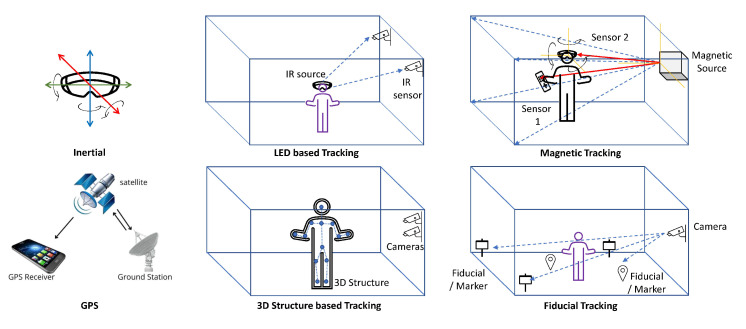
Augmented reality tracking techniques presentation.

Magnetic Tracking Technology: This technology includes a tracking source and two sensors, one sensor for the head and another one for the hand. The tracking source creates an electromagnetic field in which the sensors are placed. The computer then calculates the orientation and position of the sensors based on the signal attenuation of the field. This gives the effect of allowing a full 360 range of motion. i.e., allowing us to look all the way around the 3D environment. It also allows us to move around all three degrees of freedom. The hand tracker has some control buttons that allow the user to navigate along the environment. It allows us to pick things up and understand the size and shape of the objects [45]. In Figure 5 we have tried to draw the tracking techniques to give a better understanding to the reader.

Frikha et al. [46] introduce a new mutual occlusion problem handler. The problem of occlusion occurs when the real objects are in front of the virtual objects in the scene. The authors use a 3D positioning approach and surgical instrument tracking in an AR environment. The paradigm is introduced that is based on monocular image-based processing. The result of the experiment suggested that this approach is capable of handling mutual occlusion automatically in real-time.

One of the main issues with magnetic tracking is the limited positioning range [47]. Orientation and position can be determined by setting up the receiver to the viewer [48]. Receivers are small and light in weight and the magnetic trackers are indifferent to optical disturbances and occlusion; therefore, these have high update rates. However, the resolution magnetic field declines with the fourth power of the distance, and the strength of magnetic fields decline with the cube of the distance [49]. Therefore, the magnetic trackers have constrained working volume. Moreover, magnetic trackers are sensitive to environments around magnetic fields and the type of magnetic material used and are also susceptible to measurement jitter [50].

Magnetic tracking technology is widely used in the range of AR systems, with applications ranging from maintenance [51] to medicine [52] and manufacturing [53].

Inertial Tracking: Magnetometers, accelerometers, and gyroscopes are examples of inertial measurement units (IMU) used in inertial tracking to evaluate the velocity and orientation of the tracked object. An inertial tracking system is used to find the three rotational degrees of freedom relative to gravity. Moreover, the time period of the trackers’ update and the inertial velocity can be determined by the change in the position of the tracker.

Advantages of Inertial Tracking: It does not require a line of sight and has no range limitations. It is not prone to optical, acoustic, magnetic, and RE interference sources. Furthermore, it provides motion measurement with high bandwidth. Moreover, it has negligible latency and can be processed as fast as one desires.

Disadvantages of Inertial Tracking: They are prone to drift of orientation and position over time, but their major impact is on the position measurement. The rationale behind this is that the position must be derived from the velocity measurements. The usage of a filter could help in resolving this issue. However, the issue could while focusing on this, the filter can decrease the responsiveness and the update rate of the tracker [54]. For the ultimate correction of this issue of the drift, the inertial sensor should be combined with any other kind of sensor. For instance, it could be combined with ultrasonic range measurement devices and optical trackers.

#### 3.1.2. Vision-Based Tracking

Vision-based tracking is defined as tracking approaches that ascertain the camera pose by the use of data captured from optical sensors and as registration. The optical sensors can be divided into the following three categories:

- visible light tracking;
- 3D structure tracking;
- infrared tracking.

In recent times, vision-based tracking AR is becoming highly popular due to the improved computational power of consumer devices and the ubiquity of mobile devices, such as tablets and smartphones, thereby making them the best platform for AR technologies. Chakrabarty et al. [55] contribute to the development of autonomous tracking by integrating the CMT into IBVS, their impact on the rigid deformable targets in indoor settings, and finally the integration of the system into the Gazebo simulator. Vision-based tracking is demonstrated by the use of an effective object tracking algorithm [56] known as the clustering of static-adaptive correspondences for deformable object tracking (CMT). Gupta et al. [57] detail the comparative analysis between the different types of vision-based tracking systems.

Moreover, Krishna et al. [58] explore the use of electroencephalogram (EEG) signals in user authentication. User authentication is similar to facial recognition in mobile phones. Moreover, this is also evaluated by combining it with eye-tracking data. This research contributes to the development of a novel evaluation paradigm and a biometric authentication system for the integration of these systems. Furthermore, Dzsotjan et al. [59] delineate the usefulness of the eye-tracking data evaluated during the lectures in order to determine the learning gain of the user. Microsoft HoloLens2’s designed Walk the Graph app was used to generate the data. Binary classification was performed on the basis of the kinematic graphs which users reported of their own movement.

Ranging from smartphones to laptops and even to wearable devices with suitable cameras located in them, visible light tracking is the most commonly used optical sensor. These cameras are particularly important because they can both make a video of the real environment and can also register the virtual content to it, and thereby can be used in video see-through AR systems.

Chen et al. [60] resolve the shortcomings of the deep learning lightning model (DAM) by combining the method of transferring a regular video to a 3D photo-realistic avatar and a high-quality 3D face tracking algorithm. The evaluation of the proposed system suggests its effectiveness in real-world scenarios when we have variability in expression, pose, and illumination. Furthermore, Rambach et al. [61] explore the details pipeline of 6DoF object tracking using scanned 3D images of the objects. The scope of research covers the initialization of frame-to-frame tracking, object registration, and implementation of these aspects to make the experience more efficient. Moreover, it resolves the challenges that we faced with occlusion, illumination changes, and fast motion.

#### 3.1.3. Three-Dimensional Structure Tracking

Three-dimensional structure information has become very affordable because of the development of commercial sensors capable of accomplishing this task. It was begun after the development of Microsoft Kinect [62]. Syahidi et al. [63] introduce a 3D AR-based learning system for pre-school children. For determining the three-dimensional points in the scene, different types of sensors could be used. The most commonly used are the structured lights [64] or the time of flight [65]. These technologies work on the principle of depth analysis. In this, the real environment depth information is extracted by the mapping and the tracking [66]. The Kinect system [67], developed by Microsoft, is one of the widely used and well-developed approaches in Augmented Reality.

Rambach et al. [68] present the idea of augmented things: utilizing off-screen rendering of 3D objects, the realization of application architecture, universal 3D object tracking based on the high-quality scans of the objects, and a high degree of parallelization. Viyanon et al. [69] focus on the development of an AR app known as “AR Furniture" for providing the experience of visualizing the design and decoration to the customers. The customers fit the pieces of furniture in their rooms and were able to make a decision regarding their experience. Turkan et al. [70] introduce the new models for teaching structural analysis which has considerably improved the learning experience. The model integrates 3D visualization technology with mobile AR. Students can enjoy the different loading conditions by having the choice of switching loads, and feedback can be provided in the real-time by AR interface.

#### 3.1.4. Infrared Tracking

The objects that emitted or reflected the light are some of the earliest vision-based tracking techniques used in AR technologies. Their high brightness compared to their surrounding environment made this tracking very easy [71,72]. The self-light emitting targets were also indifferent to the drastic illumination effects i.e., harsh shadows or poor ambient lighting. In addition, these targets could either be transfixed to the object being tracked and camera at the exterior of the object and was known as “outside-looking-in” [73]. Or it could be “inside-looking-out”, external in the environment with camera attached to the target [74]. The inside-looking-out configuration, compared to the sensor of the inside-looking-out system, has greater resolution and higher accuracy of angular orientation. The inside-looking-out configuration is used in the development of several systems [20,75,76,77], typically with infrared LEDs mounted on the ceiling and a head-mounted display with a camera facing externally.

#### 3.1.5. Model-Based Tracking

The three-dimensional tracking of real-world objects has been the subject of researchers’ interest. It is not as popular as natural feature tracking or planner fiducials, however, a large amount of research has been done on it. In the past, tracking the three-dimensional model of the object was usually created by the hand. In this system, the lines, cylinders, spheres, circles, and other primitives were combined to identify the structure of objects [78]. Wuest et al. [79] focus on the development of the scalable and performance pipeline for creating a tracking solution. The structural information of the scene was extracted by using the edge filters. Additionally, for the determination of the pose, edge information and the primitives were matched [80].

In addition, Gao et al. [81] explore the tracking method to identify the different vertices of a convex polygon. This is done successfully as most of the markers are square. The coordinates of four vertices are used to determine the transformation matrix of the camera. Results of the experiment suggested that the algorithm was so robust to withstand fast motion and large ranges that make the tracking more accurate, stable, and real time.

The combination of edge-based tracking and natural feature tracking has the following advantages:

- It provides additional robustness [82].
- Enables spatial tracking and thereby is able to be operated in open environments [83].
- For variable and complex environments, greater robustness was required. Therefore, they introduced the concept of keyframes [84] in addition to the primitive model [85].

Figen et al. [86] demonstrate of a series of studies that were done at the university level in which participants were asked to make the mass volume of buildings. The first study demanded the solo work of a designer in which they had to work using two tools: MTUIs of the AR apps and analog tools. The second study developed the collaboration of the designers while using analog tools. The study has two goals: change in the behavior of the designer while using AR apps and affordances of different interfaces.

**Figure 6:**
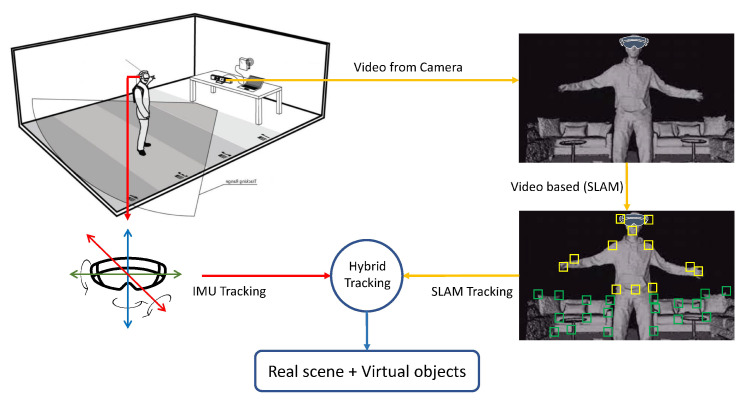
Hybrid tracking: inertial and SLAM combined and used in the latest mobile-based AR tracking.

Developing and updating the real environment’s map simultaneously had been the subject of interest in model-based tracking. This has a number of developments. First, simultaneous localization and map building (SLAM) was primarily done for robot navigation in unknown environments [87]. In augmented reality, [88,89], this technique was used for tracking the unknown environment in a drift-free manner. Second, parallel mapping and tracking [88] was developed especially for AR technology. In this, the mapping of environmental components and the camera tracks were identified as a separate function. It improved tracking accuracy and also overall performance. However, like SLAM, it did not have the capability to close large loops in the constrained environment and area (Figure 6).

Oskiper et al. [90] propose a simultaneous localization and mapping (SLAM) framework for sensor fusion, indexing, and feature matching in AR apps. It has a parallel mapping engine and error-state extended Kalman filter (EKF) for these purposes. Zhang et al.’s [91] Jaguar is a mobile tracking AR application with low latency and flexible object tracking. This paper discusses the design, execution, and evaluation of Jaguar. Jaguar enables a markerless tracking feature which is enabled through its client development on top of ARCoreest from Google. ARCore is also helpful for context awareness while estimating and recognizing the physical size and object capabilities, respectively.

#### 3.1.6. Global Positioning System—GPS Tracking

This technology refers to the positioning of outdoor tracking with reference to the earth. The present accuracy of the GPS system is up to 3 m. However, improvements are available with the advancements in satellite technology and a few other developments. Real-time kinematic (RTS) is one example of them. It works by using the carrier of a GPS signal. The major benefit of it is that it has the ability to improve the accuracy level up to the centimeter level. Feiner’s touring machine [92] was the first AR system that utilized GPS in its tracking system. It used the inclinometer/magnetometer and differential GPS positional tracking. The military, gaming [93,94], and the viewership of historical data [95] have applied GPS tracking for the AR experiences. As it only has the supporting positional tracking low accuracy, it could only be beneficial in the hybrid tracking systems or in the applications where the pose registration is not important. AR et al. [96] use the GPS-INS receiver to develop models for object motion having more precision. Ashutosh et al. [97] explore the hardware challenges of AR technology and also explore the two main components of hardware technology: battery performance and global positioning system (GPS). Table 1 provides a succinct categorization of the prominent tracking technologies in augmented reality. Example studies are referred to while highlighting the advantages and challenges of each type of tracking technology. Moreover, possible areas of application are suggested.

#### 3.1.7. Miscellaneous Tracking

Yang et al. [98], in order to recognize the different forms of hatch covers having similar shapes, propose tracking and cover recognition methods. The results of the experiment suggest its real-time property and practicability, and tracking accuracy was enough to be implemented in the AR inspection environment. Kang et al. [99] propose a pupil tracker which consists of several features that make AR more robust: key point alignment, eye-nose detection, and infrared (NIR) led. NIR led turns on and off based on the illumination light. The limitation of this detector is that it cannot be applied in low-light conditions.

**Table 1:** Summary of tracking techniques and their related attributes.

No.	Tracking Technology	Category of Tracking Technique	Status of Technique, Used in Current Devices	Tools/Company Currently Using the Technology	Key Concepts	Advantages	Challenges	Example Application Areas	Example Studies
1	Magnetic	Marker-less/Sensor based	Yes	i. Edge Tracking/Premo etc. ii. Most HMD/Most Recent Android Devices	Sensors are placedwithin an electromagnetic field	+360 degree motion +navigation around the environments +manipulationof objects	-limited positioning range -constrained working volume -highly sensitive to surrounding environments	Maintenance Medicine Manufacturing	[45,46,47,48,49,50,51,52,53]
2	Inertial	Marker-less/Sensor based	Yes	ARCore/Unity	Motion sensors (e.g., accelerometers and gyroscopes) are used to determine the velocity and orientation of objects	+high-bandwidth motion measurement +Negligible latency	-drift overtime impacting position measurement	Transport Sports	[54]
3	Optical	Marker-less/Vision based	Yes	i. Unity ii. Opti Track Used in conguction with Inertial sensors + Optical (Vision Based) sensors	Virtual content is added to real environments through cameras and optical sensors.Example approaches include visible light, 3D structure, and infrared tracking.	+Popular due to affordable consumer devices +Strong tracking algorithms +Applicationto real-world scenarios	-occlusion when objects are in close range	Education and Learning E-commerce Tourism	[100,101]
4	Model Based i. Edge-Based ii. Template-Based iii. Depth Imaging	Marker-less/Computer Vision-based	Yes	i. VisionLib ii. Unity iii. ViSP	A 3D model is visualized of real objects	+implicit knowledge of the 3D structure +empowersspatial tracking +robustness is achieved even in complex environments	-algorithms are required to track and predict movements -models need to be created using dedicated tools and libraries	Manufacturing Construction Entertainment	[78,79,80,81,82,83,84,85,86]
5	GPS	Marker-less/Sensor based	Yes	i. ARCore/ARKit ii. Unity/ARFoundation iii. Vuforia	GPS sensors are employed to track the price location of objects in the environment	+high tracking accuracy (up to cms)	-hardware requirements -objects should be modelled ahead	Gaming	[102,103,104,105,106,107]
6	Hybrid	Marker-less/Sensor based/Computer Vision	Yes	i. ARCore ii. ARKit	A mix of markerless technologies is used to overcome the challenges of a single-tracking technology	+improved tracking range and accuracy +higher degree of freedom +lower drift and jitter	-the need for multiple technologies (e.g., accelerators, sensors) so cost issues	Simulation Transport	[108,109,110,111]
7	SLAM	Marker-less/Computer Vision/Non-Model-based	Yes	i. WikiTude ii. Unity iii. ARCore	A map is created via a vision of the real environment to track the virtual object on it.	Can track unknown environments, Parallel mapping engine	Does not have the capability to close large loops in the constrained environment	Mobile based AR Tracking, Robot Navigation,	[112,113,114]
8	Structure from Motion (SFM)	Marker-Less/Computer Vision/Non-Model-Based	Yes	i. SLAM ii. Research Based	3D model reconstruction approach based on Multi View Stereo	Can be used for estimating the 3D structure of a scene from a series of 2D images	Shows limited reconstruction ability in vegetated environments	3-D scanning, augmented reality, and visual simultaneous localization and mapping (vSLAM)	[90]
9	Fiducial/Landmark	Marker-based /Fiducial	Yes	i. Solar/Unity ii. Uniducial/Unity	Tracking is made with reference to artificial landmarks (i.e., markers) added to the AR environment	+better accuracy is achieved +stable tracking with less cost	-the need for landmarks -requires image recognition (i.e., camera) -less flexible compared to marker-based	Marketing	[115,116,117]
10	QR Code based Tracking	Marker-Based/Tag-Based	Yes	Microsoft Hololense/Immersive Headsets/Unity	Tracking is made	+better accuracyis achieved +stable tracking with less cost	QR codes pose significant security risks.	Supply Chain Management	[115]

Moreover, Bach et al. [118] introduce an AR canvas for information visualization which is quite different from the traditional AR canvas. Therefore, dimensions and essential aspects for developing the visualization design for AR-canvas while enlisting the several limitations within the process. Zeng et al. [119] discuss the design and the implementation of FunPianoAR for creating a better AR piano learning experience. However, a number of discrepancies occurred with this system, and the initiation of a hybrid system is a more viable option. Rewkowski et al. [120] introduce a prototype system of AR to visualize the laparoscopic training task. This system is capable of tracking small objects and requires surgery training by using widely compatible and inexpensive borescopes.

#### 3.1.8. Hybrid Tracking

Hybrid tracking systems were used to improve the following aspects of the tracking systems:

- Improving the accuracy of the tracking system.
- Coping with the weaknesses of the respective tracking methods.
- Adding more degrees of freedom.

Gorovyi et al. [108] detail the basic principles that make up an AR by proposing a hybrid visual tracking algorithm. The direct tracking techniques are incorporated with the optical flow technique to achieve precise and stable results. The results suggested that they both can be incorporated to make a hybrid system, and ensured its success in devices having limited hardware capabilities. Previously, magnetic tracking [109] or inertial trackers [110] were used in the tracking applications while using the vision-based tracking system. Isham et al. [111] use a game controller and hybrid tracking to identify and resolve the ultrasound image position in a 3D AR environment. This hybrid system was beneficial because of the following reasons:

- Low drift of vision-based tracking.
- Low jitter of vision-based tracking.
- They had a robust sensor with high update rates. These characteristics decreased the invalid pose computation and ensured the responsiveness of the graphical updates [121].
- They had more developed inertial and magnetic trackers which were capable of extending the range of tracking and did not require the line of sight. The above-mentioned benefits suggest that the utilization of the hybrid system is more beneficial than just using the inertial trackers.

In addition, Mao et al. [122] propose a new tracking system with a number of unique features. First, it accurately translates the relative distance into the absolute distance by locating the reference points at the new positions. Secondly, it embraces the separate receiver and sender. Thirdly, resolves the discrepancy in the sampling frequency between the sender and receiver. Finally, the frequency shift due to movement is highly considered in this system. Moreover, the combination of the IMU sensor and Doppler shift with the distributed frequency modulated continuous waveform (FMCW) helps in the continuous tracking of mobile due to multiple time interval developments. The evaluation of the system suggested that it can be applied to the existing hardware and has an accuracy to the millimeter level.

The GPS tracking system alone only provides the positional information and has low accuracy. So, GPS tracking systems are usually combined with vision-based tracking or inertial sensors. The intervention would help gain the full pose estimation of 6DoF [123]. Moreover, backup tracking systems have been developed as an alternative when the GPS fails [98,124]. The optical tracking systems [100] or the ultrasonic rangefinders [101] can be coupled with the inertial trackers for enhancing efficiency. As the differential measurement approach causes the problem of drift, these hybrid systems help resolve them. Furthermore, the use of gravity as a reference to the inertial sensor made them static and bound. The introduction of the hybrid system would make them operate in a simulator, vehicle, or in any other moving platform [125]. The introduction of accelerators, cameras, gyroscopes [126], global positioning systems [127], and wireless networking [128] in mobile phones such as tablets and smartphones also gives an opportunity for hybrid tracking. Furthermore, these devices have the capability of determining outdoor as well as indoor accurate poses [129].

### 3.2. Marker-Based Tracking

Fiducial Tracking: Artificial landmarks for aiding the tracking and registration that are added to the environment are known as fiducial. The complexity of fiducial tracking varies significantly depending upon the technology and the application used. Pieces of paper or small colored LEDs were used typically in the early systems, which had the ability to be detected using color matching and could be added to the environment [130]. If the position of fiducials is well-known and they are detected enough in the scene then the pose of the camera can be determined. The positioning of one fiducial on the basis of a well-known previous position and the introduction of additional fiducials gives an additional benefit that workplaces could dynamically extend [131]. A QR code-based fudicial/marker is also proposed by some researchers for marker-/tag-based tracking [115]. With the progression of work on the concept and complexity of the fiducials, additional features such as multi-rings were introduced for the detection of fiducials at much larger distances [116]. A minimum of four points of a known position is needed for determining for calculating the pose of the viewer [117]. In order to make sure that the four points are visible, the use of these simpler fiducials demanded more care and effort for placing them in the environment. Examples of such fiducials are ARToolkit and its successors, whose registration techniques are mostly planar fiducial. In the upcoming section, AR display technologies are discussed to fulfill all the conditions of Azuma’s definition.

### 3.3. Summary

**Figure 7:**
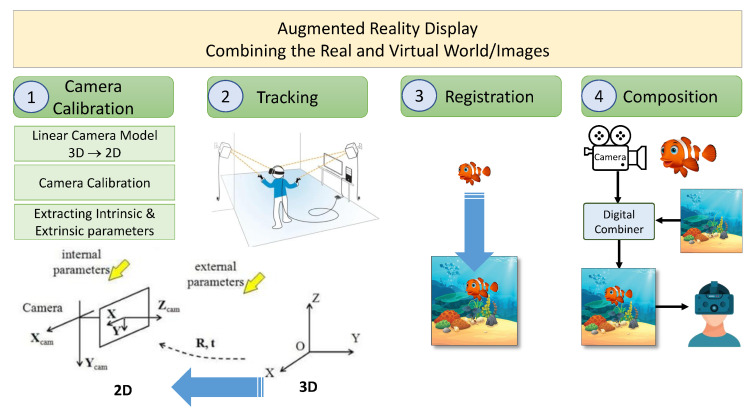
Steps for combining real and virtual content.

This section provides comprehensive details on tracking technologies that are broadly classified into markerless and marker-based approaches. Both types have many subtypes whose details, applications, pros, and cons are provided in a detailed fashion. The different categories of tracking technologies are presented in Figure 4, while the summary of tracking technologies is provided in Figure 7. Among the different tracking technologies, hybrid tracking technologies are the most adaptive. This study combined SLAM and inertial tracking technologies as part of the framework presented in the paper.

## 4. Augmented Reality Display Technology

For the combination of a real and the virtual world in such a way that they both look superimposed on each other, as in Azuma’s definition, some technology is necessarily required to display them.

### 4.1. Combination of Real and the Virtual Images

Methods or procedures required for the merging of the virtual content in the physical world include camera calibration, tracking, registration, and composition as depicted in Figure 7.

### 4.2. Camera vs. Optical See Through Calibration

It is a procedure or an optical model in which the eye display geometry or parameters define the user’s view. Or, in other words, it is a technique of complementing the dimensions and parameters of the physical and the virtual camera.

In AR, calibration can be used in two ways, one is camera calibration, and another is optical calibration. The camera calibration technique is used in video see-through (VST) displays. However, optical calibration is used in optical see-through (OST) displays. OST calibration can be further divided into three umbrellas of techniques. Initially, manual calibration techniques were used in OST. Secondly, semi-automatic calibration techniques were used, and thirdly, we have now automatic calibration techniques. Manual calibration requires a human operator to perform the calibration tasks. Semi-automatic calibration, such as simple SPAAM and display relative calibration (DRC), partially collect some parameters automatically, which usually needed to be done manually in earlier times by the user. Thirdly, the automatic OST calibration was proposed by Itoh et al. in 2014 with the model of interaction-free display calibration technique (INDICA) [132]. In video see through (VST), computer vision techniques such as cameras are used for the registration of real environments. However, in optical see through (OST), VST calibration techniques cannot be used as it is more complex because cameras are replaced by human eyes. Various calibration techniques were developed for OST. The author evaluates the registration accuracy of the automatic OST head-mounted display (HMD) calibration technique called recycled INDICA presented by Itoh and Klinker. In addition, two more calibration techniques called the single-point active alignment method (SPAAM) and degraded SPAAM were also evaluated. Multiple users were asked to perform two separate tasks to check the registration and the calibration accuracy of all three techniques can be thoroughly studied. Results show that the registration method of the recycled INDICA technique is more accurate in the vertical direction and showed the distance of virtual objects accurately. However, in the horizontal direction, the distance of virtual objects seemed closer than intended [133]. Furthermore, the results show that recycled INDICA is more accurate than any other common technique. In addition, this technique is also more accurate than the SPAAM technique. Although, different calibration techniques are used for OST and VST displays, as discussed in [133], they do not provide all the depth cues, which leads to interaction problems. Moreover, different HMDs have different tracking systems. Due to this, they are all calibrated with an external independent measuring system. In this regard, Ballestin et al. propose a registration framework for developing AR environments where all the real objects, including users, and virtual objects are registered in a common frame. The author also discusses the performance of both displays during interaction tasks. Different simple and complex tasks such as 3D blind reaching are performed using OST and VST HMDs to test their registration process and interaction of the users with both virtual and real environments. It helps to compare the two technologies. The results show that these technologies have issues, however, they can be used to perform different tasks [134].

#### Non-Geometric Calibration Method

Furthermore, these geometric calibrations lead to perceptual errors while converting from 3D to 2D [135]. To counter this problem, parallax-free video see-through HMDs were proposed; however, they were very difficult to create. In this regard, Cattari et al. in 2019 proposes a non-stereoscopic video see-through HMD for a close-up view. It mitigates perceptual errors by mitigating geometric calibration. Moreover, the authors also identify the problems of non-stereoscopic VST HMD. The aim is to propose a system that provides a view consistent with the real world [136,137]. Moreover, State et al. [138] focus on a VST HMD system that generates zero eye camera offset. While Bottechia et al. [139] present an orthoscope monocular VST HMD prototype.

### 4.3. Tracking Technologies

Some sort of technology is required to track the position and orientation of the object of interest which could either be a physical object or captured by a camera with reference to the coordinate plan (3D or 2D) of a tracking system. Several technologies ranging from computer vision techniques to 6DoF sensors are used for tracking the physical scenes.

### 4.4. Registration

Registration is defined as a process in which the coordinate frame used for manifesting the virtual content is complemented by the coordinate frame of the real-world scene. This would help in the accurate alignment of the virtual content and the physical scene.

### 4.5. Composition

Now, the accuracy of two important steps, i.e., the accurate calibration of the virtual camera and the correct registration of the virtual content relative to the physical world, signifies the right correspondence between the physical environment and the virtual scene which is generated on the basis of tracking updates. This process then leads to the composition of the virtual scene’s image and can be done in two ways: Optically (or physically) or digitally. The physical or digital composition depends upon the configuration and dimensions of the system used in the augmented reality system.

### 4.6. Types of Augmented Reality Displays

**Figure 8:**
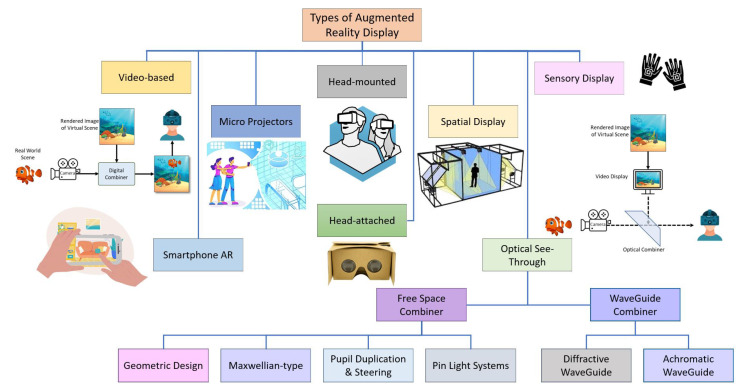
Types of augmented reality display technologies.

The combination of virtual content in the real environment divides the AR displays into four major types, as depicted in Figure 8. All have the same job to show the merged image of real and virtual content to the user’s eye. The authors have categorized the latest technologies of optical display after the advancements in holographic optical elements HOEs. There are other categories of AR display that arealso used, such as video-based, eye multiplexed, and projection onto a physical surface.

### 4.7. Optical See-Through AR Display

These kinds of displays use the optical system to merge the real scenes and virtual scene images. Examples of AR displays are head-up display HUD systems of advanced cars and cockpits of airplanes. These systems consist of the following components: beam splitters, which can be of two forms, combined prisms or half mirrors. Most beam splitters reflect the image from the video display. This reflected image is then integrated with a real-world view that can be visualized from the splitter. For half mirrors as a beam splitter, the working way is somewhat different: the real-world view is reflected on the mirror rather than the image of the video display. At the same time, the video display can also be viewed from the mirror. The transport projection system is semi-transparent optical technology used in optical display systems. Their semi-transparent property allows the viewer to witness the view at the back of the screen. Additionally, this system uses diffused light to manifest the exhibited image. Examples of semi-display optical systems are transparent projection film, transparent LCDs, etc. Optical combiners are used for the combination of virtual and real scene images. Optical see-through basically has two sub-categories, one is a free-space combiner and the other is a wave-guide combiner [140]. Additionally, now the advancement of technology has enabled technicians to make self-transparent displays. This self-transparent feature help in the miniaturization and simplification of the size and structure of the optical see-through displays.

#### 4.7.1. Free-Space Combiners

Papers related to free space combiners are discussed here. Pulli et al. [11] introduce a second-generation immersive optical see-through AR system known as meta 2. It is based on an optical engine that uses the free-form visor to make a more immersive experience. Another traditional geometric display is ultra-fast high-resolution piezo linear actuators combined with Alvarez’s lens to make a new varifocal optical see-through HMD. It uses a beamsplitter which acts as an optical combiner to merge the light paths of the real and virtual worlds [12]. Another type of free-space combiner is Maxwellian-type [112,113,114,141]. In [142], the author employs the random structure as a spatial light modulator for developing a light-field near-eye display based on random pinholes. The latest work in [143,144] introduces an Ini-based light field display using the multi-focal micro-lens to propose the extended depth of the field. To enhance the eyebox view there is another technique called puppil duplication steering [145,146,147,148,149,150]. In this regard, refs. [102,151] present the eyebox-expansion method for the holographic near-eye display and pupil-shifting holographic optical element (PSHOE) for the implementation. Additionally, the design architecture is discussed and the incorporation of the holographic optical element within the holographic display system is discussed. There is another recent technique similar to the Maxwellian view called pin-light systems. It increases the Maxwellian view with larger DoFs [103,104].

#### 4.7.2. Wave-Guide Combiner

The waveguide combiner basically traps light into TIR as opposed to free-space, which lets the light propagate without restriction [104,105,106]. The waveguide combiner has two types, one is diffractive waveguides and another is achromatic waveguides [107,152,153,154,155].

### 4.8. Video-Based AR Displays

These displays execute the digital processes as their working principle [156]. To rephrase, the merging of the physical world video and the virtual images, in video display systems, is carried out by digital processing. The working of the video-based system depends upon the video camera system by which it fabricates the real-world video into digital. The rationale behind this system is that the composition of the physical world’s video or scenario with the virtual content could be manifested digitally through the operation of a digital image processing technique [157]. Mostly, whenever the user has to watch the display, they have to look in the direction of the video display, and the camera is usually attached at the back of this display. So, the camera faces the physical world scene. These are known as “video see-through displays" because in them the real world is fabricated through the digitization (i.e., designing the digital illusion) of these video displays. Sometimes the design of the camera is done in such a way that it may show an upside-down image of an object, create the illusion of a virtual mirror, or site the image at a distant place.

### 4.9. Projection-Based AR Display

Real models [158] and walls [159] could be example of projection-based AR displays. All the other kinds of displays use the display image plan for the combination of the real and the virtual image. However, this display directly overlays the virtual scene image over the physical object. They work in the following manner:

- First, they track the user’s viewpoint.
- Secondly, they track the physical object.
- Then, they impart the interactive augmentation [160].

Mostly, these displays have a projector attached to the wall or a ceiling. This intervention has an advantage as well as a disadvantage. The advantage is that this does not demand the user to wear something. The disadvantage is that it is static and restricts the display to only one location of projection. For resolving this problem and making the projectors mobile, a small-sized projector has been made that could be easily carried from one place to another [161]. More recently, with the advancement of technology, miniaturized projectors have also been developed. These could be held in the hand [162] or worn on the chest [163] or head [164].

### 4.10. Eye-Multiplexed Augmented Reality Display

In eye-multiplexed AR displays, the users are allowed to combine the views of the virtual and real scenes mentally in their minds [72]. Rephrased, these displays do not combine the image digitally; therefore, it requires less computational power [72]. The process is as follows. First, the virtual image gets registered to the physical environment. Second, the user will get to see the same rendered image as the physical scene because the virtual image is registered to the physical environment. The user has to mentally configure the images in their mind to combine the virtual and real scene images because the display does not composite the rendered and the physical image. For two reasons, the display should be kept near the viewer’s eye: first, the display could appear as an inset into the real world, and second, the user would have to put less effort into mentally compositing the image.

The division of the displays on the basis of the position of the display between the real and virtual scenes is referred to as the “eye to world spectrum”.

### 4.11. Head-Attached Display

Head-attached displays are in the form of glasses, helmets, or goggles. They vary in size from smaller to bigger. However, with the advancement of technology, they are becoming lighter to wear. They work by displaying the virtual image right in front of the user’s eye. As a result, no other physical object can come between the virtual scene and the viewer’s eye. Therefore, the third physical object cannot occlude them. In this regard, Koulieris et al. [165] summarized the work on immersive near-eye tracking technologies and displays. Results suggest various loopholes within the work on display technologies: user and environmental tracking and emergence–accommodation conflict. Moreover, it suggests that advancement in the optics technology and focus adjustable lens will improve future headset innovations and creation of a much more comfortable HMD experience. In addition to it, Minoufekr et al. [166] illustrate and examine the verification of CNC machining using Microsoft HoloLens. In addition, they also explore the performance of AR with machine simulation. Remote computers can easily pick up the machine models and load them onto the HoloLens as holograms. A simulation framework is employed that makes the machining process observed prior to the original process. Further, Franz et al. [88] introduce two sharing techniques i.e., over-the-shoulder AR and semantic linking for investigating the scenarios in which not every user is wearing HWD. Semantic linking portrays the virtual content’s contextual information on some large display. The result of the experiment suggested that semantic linking and over-the-shoulder suggested communication between participants as compared to the baseline condition. Condino et al. [167] aim to explore two main aspects. First, to explore complex craniotomies to gauge the reliability of the AR-headsets [168]. Secondly, for non-invasive, fast, and completely automatic planning-to-patient registration, this paper determines the efficacy of patient-specific template-based methodology for this purpose.

### 4.12. Head-Mounted Displays

The most commonly used displays in AR research are head-mounted displays (HMDs). They are also known as face-mounted displays or near-eye displays. The user puts them on, and the display is represented right in front of their eyes. They are most commonly in the form of goggles. While using HMDs, optical and video see-through configurations are most commonly used. However, recently, head-mounted projectors are also explored to make them small enough to wear. Examples of smart glasses, Recon Jet, Google glass, etc., are still under investigation for their usage in head-mounted displays. Barz et al. [169] introduce a real-time AR system that augments the information obtained from the recently attended objects. This system is implemented by using head-mounted displays from the state-of-the-art Microsoft HoloLens [170]. This technology can be very helpful in the fields of education, medicine, and healthcare. Fedosov et al. [171] introduce a skill system, and an outdoor field study was conducted on the 12 snowboards and skiers. First, it develops a system that has a new technique to review and share personal content. Reuter et al. [172] introduce the coordinative concept, namely RescueGlass, for German Red Cross rescue dog units. This is made up of a corresponding smartphone app and a hands-free HMD (head-mounted display) [173]. This is evaluated to determine the field of emergency response and management. The initial design is presented for collaborative professional mobile tasks and is provided using smart glasses. However, the evaluation suggested a number of technical limitations in the research that could be covered in future investigations. Tobias et al. [174] explore the aspects such as ambiguity, depth cues, performed tasks, user interface, and perception for 2D and 3D visualization with the help of examples. Secondly, they categorize the head-mounted displays, introduce new concepts for collaboration tasks, and explain the concepts of big data visualization. The results of the study suggested that the use of collaboration and workspace decisions could be improved with the introduction of the AR workspace prototype. In addition, these displays have lenses that come between the virtual view and the user’s eye just like microscopes and telescopes. So, the experiments are under investigation to develop a more direct way of viewing images such as the virtual retinal display developed in 1995 [175]. Andersson et al. [176] show that training, maintenance, process monitoring, and programming can be improved by integrating AR with human—robot interaction scenarios.

### 4.13. Body-Attached and Handheld Displays

Previously, the experimentation with handheld display devices was done by tethering the small LSDs to the computers [177,178]. However, advancements in technology have improved handheld devices in many ways. Most importantly, they have become so powerful to operate AR visuals. Many of them are now used in AR displays such as personal digital assistants [179], cell phones [180], tablet computers [181], and ultra-mobile PCs [182].

#### 4.13.1. Smartphones and Computer tablets

In today’s world, computer tablets and smartphones are powerful enough to run AR applications, because of the following properties: various sensors, cameras, and powerful graphic processors. For instance, Google Project Tango and ARCore have the most depth imaging sensors to carry out the AR experiences. Chan et al. [183] discuss the challenges faced while applying and investigating methodologies to enhance direct touch interaction on intangible displays. Jang et al. [184] aim to explore e-leisure due to enhancement in the use of mobile AR in outdoor environments. This paper uses three methods, namely markerless, marker-based, and sensorless to investigate the tracking of the human body. Results suggested that markerless tracking cannot be used to support the e-leisure on mobile AR. With the advancement of electronic computers, OLED panels and transparent LCDs have been developed. It is also said that in the future, building handheld optical see-through devices would be available. Moreover, Fang et al. [185] focus on two main aspects of mobile AR. First, a combination of the inertial sensor, 6DoF motion tracking based on sensor-fusion, and monocular camera for the realization of mobile AR in real-time. Secondly, to balance the latency and jitter phenomenon, an adaptive filter design is introduced. Furthermore, Irshad et al. [186] introduce an evaluation method to assess mobile AR apps. Additionally, Loizeau et al. [187] explore a way of implementing AR for maintenance workers in industrial settings.

#### 4.13.2. Micro Projectors

Micro projectors are an example of a mobile phone-based AR display. Researchers are trying to investigate these devices that could be worn on the chest [188], shoulder [189], or wrist [190]. However, mostly they are handheld and look almost like handheld flashlights [191].

#### 4.13.3. Spatial Displays

Spatial displays are used to exhibit a larger display. Henceforth, these are used in the location where more users could get benefit from them i.e., public displays. Moreover, these displays are static, i.e., they are fixed at certain positions and can not be mobilized.

The common examples of spatial displays include those that create optical see-through displays through the use of optical beamers: half mirror workbench [192,193,194,195] and virtual showcases. Half mirrors are commonly used for the merging of haptic interfaces. They also enable closer virtual interaction. Virtual showcases may exhibit the virtual images on some solid or physical objects mentioned in [196,197,198,199,200]. Moreover, these could be combined with the other type of technologies to excavate further experiences. The use of volumetric 3D displays [201], autostereoscopic displays [202], and other three-dimensional displays could be researched to investigate further interesting findings.

#### 4.13.4. Sensory Displays

In addition to visual displays, there are some sensors developed that work with other types of sensory information such as haptic or audio sensors. Audio augmentation is easier than video augmentation because the real world and the virtual sounds get naturally mixed up with each other. However, the most challenging part is to make the user think that the virtual sound is spatial. Multi-channel speaker systems and the use of stereo headphones with the head-related transfer function (HRTF) are being researched to cope with this challenge [203]. Digital sound projectors use the reverberation and the interference of sound by using a series of speakers [204]. Mic-throughand hear-through systems, developed by Lindeman [205,206,206], work effectively and are analogous to video and optical see-through displays. The feasibility test for this system was done by using a bone conduction headset. Other sensory experiences are also being researched. For example, the augmentation of the gustatory and olfactory senses. Olfactory and visual augmentation of a cookie-eating scene was developed by Narumi [207]. Table 2 gives the primary types of augmented reality display technologies and discusses their advantages and disadvantages.

### 4.14. Summary

This section presented a comprehensive survey of AR display technologies. These displays not only focused on combing the virtual and real-world scenes of visual experience but also other ways of combining the sensory, olfactory, and gustatory senses are also under examination by researchers. Previously, head-mounted displays were most commonly in practice; however, now handheld devices and tablets or mobile-based experiences are widely used. These things may also change in the future depending on future research and low cost. The role of display technologies was elaborated first, thereafter, the process of combining the real and augmented contents and visualizing these to users was elaborated. The section elaborated thoroughly on where the optical see-through and video-based see-through are utilized along with details of devices. Video see-through (VST) is used in head-mounted displays and computer vision techniques such as cameras are used for registration of real environment, while in optical see-through (OST), VST calibration techniques cannot be used due to complexity, and cameras are replaced by human eyes. The optical see-through is a trendy approach as of now. The different calibration approaches are presented and analyzed and it is identified after analysis, the results show that recycled INDICA is more accurate than other common techniques presented in the paper. This section also presents video-based AR displays. Figure 8 present a classified representation of different display technologies pertaining to video-based, head-mounted, and sensory-based approaches. The functions and applications of various display technologies are provided in Table 2 Each of the display technologies presented has its applicability in various realms whose details are summarized in the same Table 2.

## 5. Walking and Distance Estimation in AR

The effectiveness of AR technologies depends on the perception of distance of users from both real and virtual objects [214,215]. Mikko et al. performed some experiments to judge depth using stereoscopic depth perception [216]. The perception can be changed if the objects are on the ground or off the ground. In this regard, Carlos et al. also proposed a comparison between the perception of distance of these objects on the ground and off the ground. The experiment was done where the participant perceived the distance from cubes on the ground and off the ground as well. The results showed that there is a difference between both perceptions. However, it was also shown that this perception depends on whether the vision is monocular or binocular [217]. Plenty of research has been done in outdoor navigation and indoor navigation areas with AR [214]. In this regard, Umair et al. present an indoor navigation system in which Google glass is used as a wearable head-mounted display. A pre-scanned 3D map is used to track an indoor environment. This navigation system is tested on both HMD and handheld devices such as smartphones. The results show that the HMD was more accurate than the handheld devices. Moreover, it is stated that the system needs more improvement [218].

## 6. AR Development Tool

**Figure 9:**
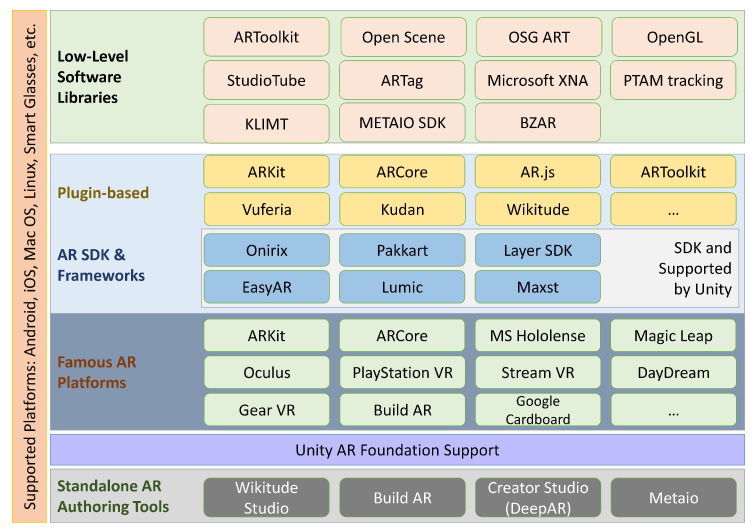
Stack of development libraries, plug-ins, platforms, and standalone authoring tools for augmented reality development.

In addition to the tracking and display devices, there are some other software tools required for creating an AR experience. As these are hardware devices, they require some software to create an AR experience. This section explores the tools and the software libraries. It will cover both the aspects of the commercially available tools and some that are research related. Different software applications require a separate AR development tool. A complete set of low-level software libraries, plug-ins, platforms, and standalones are presented in Figure 9 so they can be summarized for the reader.

In some tools, computer vision-based tracking (see Section 3.1.2) is preferred for creating an indoor experience, while others utilized sensors for creating an outdoor experience. The use of each tool would depend upon the type of platform (web or mobile) for which it is designed. Further in the document, the available AR tools are discussed, which consist of both novel tools and those that are widely known. Broadly, the following tools will be discussed:

- Low-level software development tools: needs high technological and programming skills.
- Rapid prototyping: provides a quick experience.
- Plug-ins that run on the existing applications.
- Standalone tools that are specifically designed for non-programmers.
- Next generation of AR developing tools.

### 6.1. Low-Level Software Libraries and Frameworks

Low-level software and frameworks make the functions of display and core tracking accessible for creating an AR experience. One of the most commonly used AR software libraries, as discussed in the previous section, is ARToolKit. ARToolKit is developed by Billing Hurst and Kato that has two versions [219]. It works on the principle of a fiducial marker-based registration system [220]. There are certain advances in the ARToolKit discussed related to the tracking in [213,221,222,223,224]. The first one is an open-source version that provides the marker-based tracking experience, while the second one provides natural tracking features and is a commercial version. It can be operated on Linux, Windows, and Mac OS desktops as it is written in the C language. It does not require complex graphics or built-in support for accomplishing its major function of providing a tracking experience, and it can operate simply by using low-level OpenGL-based rendering. ARToolKit requires some additional libraries such as osgART and OpenScene graph library so it can provide a complete AR experience to AR applications. OpenScene graph library is written in C language and operates as an open-source graph library. For graphic rendering, the OpenScene graph uses OpenGL. Similarly, the osgART library links the OpenScene graph and ARToolKit. It has advanced rendering techniques that help in developing the interacting AR application. OsgART library has a modular structure and can work with any other tracking library such as PTAM and BazAR, if ARtoolkit is not appropriate. BazAR is a workable tracking and geometric calibration library. Similarly, PTAM is a SLAM-based tracking library. It has a research-based and commercial license. All these libraries are available and workable to create a workable AR application. Goblin XNA [208] is another platform that has the components of interactions based on physics, video capture, a head-mounted AR display on which output is displayed, and a three-dimensional user interface. With Goblin XNA, existing XNA games could be easily modified [209]. Goblin XNA is available as a research and educational platform. Studierstube [210] is another AR system through which a complete AR application can be easily developed. It has tracking hardware, input devices, different types of displays, AR HMD, and desktops. Studierstube was specially developed to subsidize the collaborative applications [211,212]. Studierstube is a research-oriented library and is not available as commercial and workable easy-to-use software. Another commercially available SDK is Metaio SDK [225]. It consists of a variety of AR tracking technologies including image tracking, marker tracking, face tracking, external infrared tracking, and a three-dimensional object tracking. However, in May 2015, it was acquired by Apple and Metaio products and subscriptions are no longer available for purchase. Some of these libraries such as Studierstube and ARToolKit were initially not developed for PDAs. However, they have been re-developed for PDAs [226]. It added a few libraries in assistance such as open tracker, pocketknife for hardware abstraction, KLIMT as mobile rendering, and the formal libraries of communication (ACE) and screen graphs. All these libraries helped to develop a complete mobile-based AR collaborative experience [227,228]. Similarly, ARToolKit also incorporated the OpenScene graph library to provide a mobile-based AR experience. It worked with Android and iOS with a native development kit including some Java wrapping classes. Vuforia’s Qualcomm low-level library also provided an AR experience for mobile devices. ARToolKit and Vuforia both can be installed as a plug-in in Unity which provides an easy-to-use application development for various platforms. There are a number of sensors and low-level vision and location-based libraries such as Metaio SDK and Droid which were developed for outdoor AR experience. In addition to these low-level libraries, the Hit Lab NZ Outdoor AR library provided high-level abstraction for outdoor AR experience [229]. Furthermore, there is a famous mobile-based location AR tool that is called Hoppala-Augmentation. The geotags given by this tool can be browsed by any of the AR browsers including Layar, Junaio, and Wikitude [230].

### 6.2. ARTag

ARTag is designed to resolve the limitations of ARToolkit. This system was developed to resolve a number of issues:

- Resolving inaccurate pattern matching by preventing the false positive matches.
- Enhancing the functioning in the presence of the imbalanced lightening conditions.
- Making the occlusion more invariant.

However, ARTag is no longer actively under development and supported by the NRC Lab. A commercial license is not available.

### 6.3. Wikitude Studio

This is also a web-based authoring tool for creating mobile-based AR applications. It allows the utilization of computer vision-based technology for the registration of the real world. Several types of media such as animation and 3D models can be used for creating an AR scene. One of the important features of Wikitude is that the developed mobile AR content can be uploaded not only on the Wikitude AR browser app but also on a custom mobile app [231]. Wikitude’s commercial plug-in is also available in Unity to enhance the AR experience for developers.

### 6.4. Standalone AR Tools

Standalone AR tools are mainly designed to enable non-programmer users to create an AR experience. A person the basic computer knowledge can build and use them. The reason lies in the fact that most AR authoring tools are developed on a graphical user interface. It is known as a standalone because it does not require any additional software for its operation. The most common and major functions of standalone are animation, adding interactive behaviors, and construction. The earliest examples of the standalone tools are AMIRE [232] and CATOMIR [233]. However, AMIRE and CATOMIR have no support available and are not maintained by the development team.

#### BuildAR

This standalone AR authoring tool has the advantage of quickly adding to the development of the AR experience. BuildAR has important characteristics. This allows the user to add video, 3D models, sound, text, and images. It has both arbitrary images and the square marker for which it provides computer vision-based tracking. They use the format of proprietary file format for saving the content developed by the user. BuildAR viewer software can be downloaded for free and it helps in viewing the file. However, BuildAR has no support available and the exe file is not available on their website.

Limitation: It does not support adding new interactive features. However, Choi et al. [234] have provided a solution to this constraint. They have added the desktop authoring tool that helps in adding new interactive experiences.

### 6.5. Rapid Prototyping/Development Tools

In order to cope with the limitation of low-level libraries, another more fast and more rapid AR application development tool is required. The major idea behind the development of rapid prototyping was that it rapidly shows the user the prototype before executing the hard exercise of developing the application. In the following paragraphs, a number of different tools are explained for developing rapid prototyping. For the creation of multimedia content, Adobe Flashis one of the most famous tools. It was developed on desktop and web platforms. Moreover, the web desktop and mobile experiences can be prototyped by it. Flash developers can use the FLARManager, FLARToolKit, or any other plug-ins for the development of AR experience. Porting the version of ARToolKit over the flash on the web creates the AR experience. Its process is so fast that just by writing a few lines, the developer can:

- Activate their camera.
- The AR markers could be viewed in a camera.
- The virtual content could be overlaid and loaded on the tracked image.

FLARToolkit is the best platform for creating AR prototyping because it has made it very easy for being operated by anyone. Anyone who has a camera and flash-enabled web browser can easily develop the AR experience. Alternatives to Flash: According to the website of Adobe, it no longer supports Flash Player after 31 December 2020 and blocked Flash content from running in Flash Player beginning 12 January 2021. Adobe strongly recommends all users immediately uninstall Flash Player to help protect their systems. However, some AR plug-ins could be used as an alternative to Flash-based AR applications. For instance, Microsoft Silverlight has the SLARToolKit. HTML5 is also recently used by researchers for creating web-based AR experiences. The major benefit of using HTML5 is that the interference of the third-party plug-in is not required. For instance, the AR natural feature tracking is implemented on WebGL, HTML5, and JavaScript. This was developed by Oberhofer and was viewable on mobile web browsers and desktops. Additionally, the normal HTML, with few web component technologies, has been used by Ahn [235] to develop a complete mobile AR framework.

### 6.6. Plug-ins to Existing Developer Tools

For the creation of AR experiences, the software libraries require tremendous programming techniques. So, plug-ins could be used as an alternative. Plug-ins are devices that could be plugged into the existing software packages. The AR functionality is added to the software packages that to the existing two-dimensional or three-dimensional content authoring tools. If the user already knows the procedure of using authoring tools that are supported by plug-ins, then AR plug-ins for the non-AR authoring tools are useful. These tools are aimed at:

- AR tracking and visualization functions for the existing authoring tools.
- It depends on the content authoring function supplied by the main authoring tool.

There are certain tools available as plug-ins and standalone through which AR applications can be built comparatively simply. These are commercial and some of them are freely available. As discussed earlier, Vuforia can be installed as a plug-in in Unity [236] and also has a free version. However, with complete support of tools certain amount needs to be paid. Similarly, ARtoolkit is available standalone and a plug-in for Unity is available. It is freely available for various platforms such as Android, iOS, Linux, and Windows. Moreover, ARCore and ARKit are also available for Android and iOS, respectively, and can work with Unity and Unreal authoring tools as a plug-in. ARCore is available and free for developers. MAXST and Wikitude also can work in integration with Unity, though they have a licensing price for the commercial version of the software. MAXST had a free version as well. All these tools, the abovementioned libraries, and standalone tools are depicted in Figure 9. Cinema 4D, Maya, Trimble SketchUp 3D modeling software, 3Ds Max, and many others were created by a number of plug-ins that acted as authoring tools for three-dimensional content. While 3D animation and modeling tools are not capable of providing interactive features, it is very productive in creating three-dimensional scenes. SketchUp can utilize the AR plug-in by creating a model for the content creators. This model is then viewable in the AR scene provided by a free AR media player. The interactive three-dimensional graphic authoring tools are also available for the creation of highly interactive AR experiences, for instance, Wizard [237], Quest3D [238], and Unity [236]. All of these authoring tools have their own specific field of operation; however, Unity can be utilized to create a variety of experiences. The following are examples that justify the use of Unity over different solutions available:

- The AR plug-in of the Vuforia tracking library can be used with Unity 3D. This integration will help Vuforia in the creation of AR applications for the android or iOS platform.
- Similarly, the ARToolkit for Unity also provides marker-based experiences. It provides both image and marker-based AR visualization and tracking.

In such integrations, the highly interactive experiences are created by the normal Unity3D scripting interface and visual programming. Limitations of AR plug-ins: The following are the limitations accrued with the AR plug-in:

- The need for proprietary software could arise for the content produced by the authoring tool. The design provided by the authoring tools could restrict the user’s interactive and interface designs.
- Moreover, the authoring tools can also restrict the configurations of hardware or software within a certain limit.

Moreover, Nebeling et al. [239] reviewed the issues with the authoring tools of AR/VR. The survey of the tools has identified three key issues. To make up for those limitations, new tools are introduced for supporting the gesture-based interaction and rapid prototyping of the AR/VR content. Moreover, this is done without having technical knowledge of programming, gesture recognition, and 3D modeling. Mladenov et al. [240] review the existing SDKs and aim to find the most efficient SDK for the AR applications used in industrial environments. The paper reveals that currently available SDKs are very helpful for users to create AR applications with the parameters of their choice in industrial settings.

### 6.7. Summary

This section presents a detailed survey of different software and tools required for creating an AR experience. The section outlines hardware devices used in AR technology and various software to create an AR experience. It further elaborates on the software libraries required and covers bother the aspects of the commercially available tools. Table 3 provides a stack of software libraries, plug-ins, supported platforms, and standalone authoring tools. The figure also presents details of whether the mentioned tools are active or inactive. As an example, BazAR is used in tracking and geometric calibration. It is an open-source library for Linux or windows available under research-based GPL and can be used for research to detect an object via camera, calibrate it, and initiate tracking to put a basic virtual image on it; however, this library is not active at the present. Commercially used AR tools such as plug-ins have the limitations of only working efficiently in the 2D GUI and become problematic when used for 3D content. The advancement of technology may bring about a change in the authoring tools by making them capable of being operated for 3D and developing more active AR interfaces.

## 7. Collaborative Research on Augmented Reality

**Figure 10:**
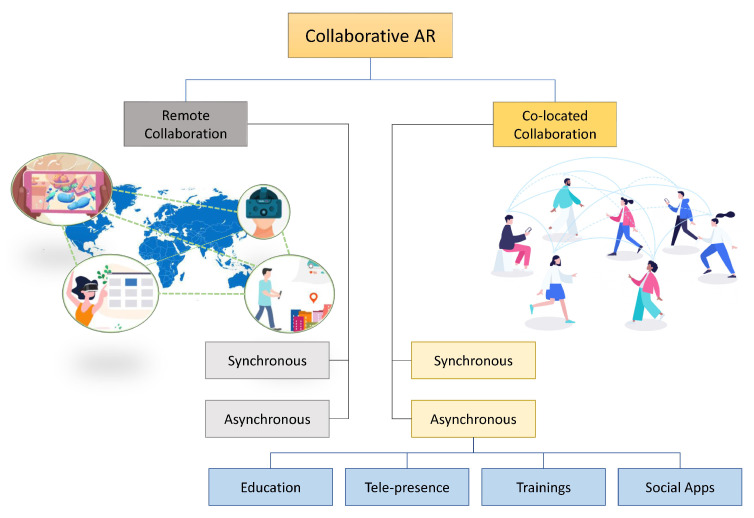
Collaborative augmented reality research domains.

In general, collaboration in augmented reality is the interaction of multiple users with virtual objects in the real environment. This interaction is regardless of the users’ location, i.e., they can participate remotely or have the same location. In this regard, we have two types of collaborative AR: co-located collaborative AR and remote collaborative AR. We mention it further in Figure 10.

### 7.1. Co-Located Collaborative AR

In this type of collaborative AR, the users interact with the virtual content rendered in the real environment while sharing the same place. The participant are not remote in such case. In this regard, Wells et al. [241] aim to determine the impact on the co-located group activities by varying the complexity of AR models using mobile AR. The paper also discusses different styles of collaborative AR such as:

- hlActive Discussion: A face-to-face discussion including all participants.
- Single Shared view: The participants focus on a single device.
- Disjoint and Shared View: Two to three participants focus on a single device.
- Disjoint and Distributed View: One to two people focus on their devices while the others are discussing.
- Distributed View: Participants focus on their devices with no discussion.
- Distributive View with Discussion: Participants focus on their devices while discussing in the group.

In this paper, the author did not contribute to the technology of co-located collaborative AR, but rather performed analysis on the effectiveness of different collaborative AR.

Grandi et al. [242] target the development of design approaches for synchronous collaboration to resolve complex manipulation tasks. For this, purpose fundamental concepts of design interface, human collaboration, and manipulation are discussed. This research the spiral model of research methodology which involves the development, planning, analysis, and evaluation. In addition, Dong et al. [243] introduce “ARVita”, a system where multiple users can interact with virtual simulations of engineering processes by wearing a head-mounted display. This system uses a co-located AR technique where the users are sitting around a table.

#### 7.1.1. Applications of Co-located Collaborative AR

Kim et al. [244] propose a PDIE model to make a STEAM educational class while incorporating AR technology into the system. Furthermore, the “Aurasma” application is used to promote AR in education. In addition, Kanzanidis et al. [245] focus on teaching mobile programming using synchronous co-located collaborative AR mobile applications in which students are distributed in groups. The result showed that the students were satisfied with this learning methodology. Moreover, Chang et al. [246] explore the use of a mobile AR (MAR) application to teach interior design activities to students. The results identified that the students who were exposed to MAR showed more effectiveness in learning than those who were taught traditionally. Lastly, Sarkar et al. [247] discuss three aspects of synchronous co-located collaboration-based problem-solving: first, students’ perspectives on AR learning activities, either in dyads or individually are determined; second, the approach adopted by students while problem-solving is determined; third, the students’ motivation for using ScholAR is determined. Statistical results suggested that 90.4% students preferred the collaborative AR experience, i.e., in dyads. Meanwhile, motivation level and usability scores were higher for individual experiences. Grandi et al. [248] introduce the design for the collaborative manipulation of AR objects using mobile AR. This approach has two main features. It provides a shared medium for collaboration and manipulation of 3D objects as well as provides precise control of DoF transformations. Moreover, strategies are presented to make this system more efficient for users in pairs. Akccayir et al. [249] explore the impact of AR on the laboratory work of university students and their attitudes toward laboratories. This study used the quasi-experimental design with 76 participants—first year students aged 18–20 years. Both qualitative and quantitative methods were used for the analyses of data. A five-week implementation of the experiment proved that the use of AR in the laboratory significantly improved the laboratory skills of the students. However, some teachers and students also discussed some of the negative impacts of other aspects of AR. Rekimoto et al. [250] propose a collaborative AR system called TransVision. In this system, two or more users use a see-through display to look at the virtual objects rendered in a real environment using synchronous co-located collaborative AR. Oda et al. [251] propose a technique for avoiding interference for hand-held synchronous co-located collaborative AR. This study is based on first-person two-player shooting AR games. Benko et al. [87] present a collaborative augmented reality and mixed reality system called “VITA” or “Visual Interaction Tool For Archaeology”. They have an off-site visualization system that allows multiple users to interact with a virtual archaeological object. Franz et al. [88] present a system of collaborative AR for museums in which multiple users can interact in a shared environment. Huynh et al. [252] introduce art of defense (AoD), a co-located augmented reality board game that combines handheld devices with physical game pieces to create a unique experience of a merged physical and virtual game. Nilsson et al. [253] focus on a multi-user collaborative AR application as a tool for supporting collaboration between different organizations such as rescue services, police, and military organizations in a critical situation.

#### 7.1.2. Asynchronous Co-Located Collaborative AR

Tseng et al. [254] present an asynchronous annotation system for collaborative augmented reality. This system can attribute virtual annotations with the real world due to a number of distinguishing capabilities, i.e., playing back, placing, and organizing. Extra context information is preserved by the recording of the perspective of the annotator. Furthermore, Kashara et al. [255] introduce “Second Surface”, an asynchronous co-located collaborative AR system. It allows the users to render images, text, or drawings in a real environment. These objects are stored in the data server and can be accessed later on.

### 7.2. Remote Collaborative AR

In this type of collaborative AR, all the users have different environments. They can interact with virtual objects remotely from any location. A number of studies have been done in this regard. Billinghurst et al. [256] introduce a wearable collaborative augmented reality system called “WearCom” to communicate with multiple remote people. Stafford et al. [257] present God-like interaction techniques for collaboration between outdoor AR and indoor tabletop users. This paper also describes a series of applications for collaboration. Gauglitz et al. [258] focus on a touchscreen interface for creating annotations in a collaborative AR environment. Moreover, this interface is also capable of virtually navigating a scene reconstructed live in 3D. Boonbrahm et al. [259] aim to develop a design model for remote collaboration. The research introduces the multiple marker technique to develop a very stable system that allows users from different locations to collaborate which also improves the accuracy. Li et al. [260] suggest the viewing of a collaborative exhibit has been considerably improved by introducing the distance-driven user interface (DUI). Poretski et al. [261] describe the behavioral challenges faced in interaction with virtual objects during remote collaborative AR. An experiment was performed to study users’ interaction with shared virtual objects in AR. Clergeaud et al. [262] tackle the limitations of collaboration in aerospace industrial designs. In addition, the authors propose prototype designs to address these limitations. Oda et al. [263] present the GARDEN (gesturing in an augmented reality depth-mapped environment) technique for 3D referencing in a collaborative augmented reality environment. The result shows that this technique is more accurate than the other comparisons. Muller et al. [85] investigate the influence of shared virtual landmarks (SVLs) on communication behavior and user experience. The results show that enhancement in user experience when SVLs were provided. Mahmood et al. [264] present a remote collaborative system for co-presence and sharing information using mixed reality. The results show improvements in user collaborative analysis experience.

#### 7.2.1. Applications of Remote Collaborative AR

Munoz et al. [265] present a system called GLUEPS-AR to help teachers in learning situations by integrating AR and web technologies i.e., Web 2.0 tools and virtual learning environments (VLEs) [266]. Bin et al. [267] propose a system to enhance the learning experience of the students using collaborative mobile augmented reality learning application (CoMARLA). The application was used to teach ICT to students. The results showed improvement in the learning of the students using CoMARLA. Dunleavy et al. [268] explore the benefits and drawbacks of collaborative augmented reality simulations in learning. Moreover, a collaborative AR system was proposed for computers independent of location, i.e., indoor or outdoor. Maimone et al. [269] introduce a telepresence system with real-time 3D capture for remote users to improve communication using depth cameras. Moreover, it also discusses the limitations of previous telepresence systems. Gauglitz et al. [270] present an annotation-based remote collaboration AR system for mobiles. In this system, the remote user can explore the scene regardless of the local user’s camera position. Moreover, they can also communicate through annotations visible on the screen. Guo et al. [271] introduce an app, known as Block, that enables the users to collaborate irrespective of their geographic position, i.e., they can be either co-located or remote. Moreover, they can collaborate either asynchronously or synchronously. This app allows users to create structures that persist in the real environment. The result of the study suggested that people preferred synchronous and collocated collaboration, particularly one that was not restricted by time and space. Zhang et al. [272] propose a collaborative augmented reality for socialization app (CARS). This app improves the user’s perception of the quality of the experience. CARS benefits the user, application, and system on various levels. It reduces the use of computer resources, end-to-end latency, and networking. Results of the experiment suggest that CARS acts more efficiently for users of cloud-based AR applications. Moreover, on mobile phones, it reduces the latency level by up to 40%. Grandi et al. [242] propose an edge-assisted system, known as CollabAR, which combines both collaboration image recognition and distortion tolerance. Collaboration image recognition enhances recognition accuracy by exploiting the “spatial-temporal" correlation. The result of the experiment suggested that this system has significantly decreased the end-to-end system latency up to 17.8 ms for a smartphone. Additionally, recognition accuracy for images with stronger distortions was found to be 96%.

#### 7.2.2. Synchronous Remote Collaborative AR

Lien et al. [273] present a system called “Pixel-Point Volume Segmentation” in collaborative AR. This system is used for object references. Moreover, one user can locate the objects with the help of circles drawn on the screen by other users in a collaborative environment. Huang et al. [274] focus on sharing hand gestures and sketches between a local user and a remote user by using collaborative AR. The system is named “HandsinTouch”. Ou et al. [275] present the DOVE (drawing over video environment) system, which integrates live-video and gestures in collaborative AR. This system is designed to perform remote physical tasks in a collaborative environment. Datcu et al. [276] present the creation and evaluation of the handheld AR system. This is done particularly to investigate the remote forensic and co-located and to support team-situational awareness. Three experienced investigators evaluated this system in two steps. First, it was investigated with one remote and one local investigator. Secondly, with one remote and two local investigators. Results of the study suggest the use of this technology resolves the limitation of HMDs. Tait et al. [277] propose the AR-based remote collaboration that supports view independence. The main aim of the system was to enable the remote user to help the local user with object placement. The remote user uses a 3D reconstruction of the environment to independently find the local user’s scene. Moreover, a remote user can also place the virtual cues in the scene visible to the local user. The major advantage of this system is that it allows the remote user to have an independent scene in the shared task space. Fang et al. [278] focus on enhancing the 3D feel of immersive interaction by reducing communication barriers. WebRTC, a real-time video communication framework, is developed to enable the operator site’s first-hand view of the remote user. Node.js and WebSocket, virtual canvas-based whiteboards, are developed which are usable in different aspects of life. Mora et al. [279] explain the CroMAR system. The authors aim to help the users in crowd management who are deployed in a planned outdoor event. CroMAR allows the users to share viewpoints via email, and geo-localized tags allow the users to visualize the outdoor environment and rate these tags. Adcock et al. [280] present three remote spacial augmented reality systems “Composite Wedge”, “Vector Box”, and “Eyelight” for off-surface 3D viewpoints visualization. In this system, the physical world environment of a remote user can be seen by the local user. Lincoln et al. [281] focus on a system of robotic avatars of humans in a synchronous remote collaborative environment. It uses cameras and projectors to render a humanoid animatronic model which can be seen by multiple users. This system is called “Animatronic Shader Lamps Avatars”. Komiyama et al. [282] present a synchronous remote collaborative AR system. It can transition between first person and third person view during collaboration. Moreover, the local user can observe the environment of the remote user. Lehment et al. [283] present an automatically aligned videoconferencing AR system. In this system, the remote user is rendered and aligned on the display of the local user. This alignment is done automatically regarding the local user’s real environment without modifying it. Oda et al. [284] present a remote collaborative system for guidance in a collaborative environment. In this system, the remote expert can guide a local user with the help of both AR and VR. The remote expert can create virtual replicas of real objects to guide a local user. Piumsomboon et al. [285] introduce an adaptive avatar system in mixed reality (MR) called “Mini Me” between a remote user using VR and a local user using AR technology. The results show that it improves the overall experience of MR and social presence. Piumsomboon et al. [286] present “CoVAR”, a collaboration consisting of both AR and VR technologies. A local user can share their environment with a remote VR user. It supports gestures, head, and eye gaze to improve the collaboration experience. Teo et al. [287] present a system that captures a 360 panorama video of one user and shares it with the other remote user in a mixed reality collaboration. In this system, the users communicate through hand gestures and visual annotation. Thanyadit et al. [288] introduce a system where the instructor can observe students in a virtual environment. The system is called “ObserVAR” and uses augmented reality to observe students’ gazes in a virtual environment. Results show that this system is more improved and flexible in several scenarios. Sodhi et al. [289] present a synchronous remote collaborative system called “BeThere” to explore 3D gestures and spatial input. This system enables a remote user to perform virtual interaction in the local user’s real environment. Ong et al. [290] propose a collaborative system in which 3D objects can be seen by all the users in a collaborative environment. Moreover, the changes made to these objects are also observed by the users. Butz et al. [84] present EMMIE (environment management for multi-user information environments) in a collaborative augmented reality environment in which virtual objects can be manipulated by the users. In addition, this manipulation is visible to each user of this system.

#### 7.2.3. Asynchronous Remote Collaborative AR

Irlitti et al. [291] explore the challenges faced during the use of asynchronous collaborative AR. Moreover, the author further discusses how to enhance communication while using asynchronous collaborative AR. Quasi-systems do not fulfill Azuma’s [292] definition of AR technology. However, they are very good at executing certain aspects of AR as other full AR devices are doing. For instance, mixed-space collaborative work in a virtual theater [268]. This system explained that if someone wants two groups to pay attention to each other, a common spatial frame of reference should be created to have a better experience of social presence. In the spatially aware educational system, students were using location-aware smartphones to resolve riddles. This was very useful in the educational system because it supported both engagement and social presence [245,265,269]. However, this system did not align the 3D virtual content in the virtual space. Therefore, it was not a true AR system. In order to capture a remote 3D scene, Fuchs and Maimone [293] developed an algorithm. They also developed a proof of concept for teleconferencing. For capturing images, RGB-D cameras were used. The remote scene was displayed on the 3D stereoscopic screen. These systems were not fully AR, but they still exhibited a very good immersion. Akussah et al. [294] focus on developing a marker-based collaborative augmented reality app for learning mathematics. First, the system focuses on individual experience and later on expands it to collaborative AR.

### 7.3. Summary

This section provides comprehensive details on collaborative augmented reality which is broadly classified into co-located collaborative AR, where participants collaborate with each other in geographically the same location, and remote collaboration. The applications of both approaches are presented as well. Co-located collaborative AR is mostly adopted in learning realms for sharing information, for example, in museums. On the other hand, in remote collaborative AR the remote user can explore the scene regardless of the local user’s camera position. The applications of this technology are mostly found in education.

## 8. AR Interaction and Input Technologies

The interaction and input technologies are detailed in this section. There are a number of input methods that are utilized in AR technologies. First, multimode and 3D interfaces such as speech, gesture and handheld wands. Second, the mouse, and keyboard traditional two-dimensional user interfaces (UI). The type of interaction task needed for the interface defines which input method would be utilized in the application. A variety of interfaces have been developed: three-dimensional user interfaces, tangible user interfaces, multimedia interfaces, natural user interfaces, and information browsers.

### 8.1. AR Information Browsers

Wikitude and Navicam are one of the most popular examples of AR information browsers. The only problem with AR browsers is that they cannot provide direct interaction with the virtual objects.

### 8.2. Three-Dimensional User Interfaces

A three-dimensional user interface uses the controllers for providing the interaction with virtual content. By using the traditional 3D user interface techniques, we can directly interact with the three-dimensional object in the virtual space. There are a number of 3D user interface interaction techniques as follows: **3D motion tracking sensors** are one of the most commonly used devices for AR interaction. The motion tracking sensors allow the following functions: tracking the parts of the user’s body and allow pointing as well as the manipulation of the virtual objects [295]. Haptic devices are also used for interacting with AR environments [296,297,298]. They mainly used as 3D pointing devices. In addition, they provide tactile and forces feedback. This will create the illusion of a physical object existing in the real world. Thereby, it helps in complementing the virtual experience. They are used in training, entertainment, and design-related AR applications.

### 8.3. Tangible User Interface

The tangible user interface is one of the main concepts of human–computer interface technology research. In this, the physical object is used for interaction [299]. It bridges the gap between the physical and the virtual object [300]. Chessa et al. incorporated grasping behavior in a virtual reality systems [301], while Han et al. presented and evaluated hand interaction techniques using tactile feedback (haptics) and physical grasping by mapping a real object with virtual objects [302].

### 8.4. Natural User Interfaces in AR

Recently, more accurate gesture and motion-based interactions for AR and VR applications have become extensively available due to the commercialization of depth cameras such as Microsoft Kinect and technical advances. Bare-hand interaction with a virtual object was made possible by the introduction of a depth camera. It provided physical interaction by tracking the dexterous hand motion. For instance, the physical objects and the user’s hands were recognized by the use of Kinect Camera, designed by the Microsoft HoloDesk [299]. The virtual objects were shown on the optical see-through AR workbench. It also allowed the users to interact with the virtual objects presented on the AR workbench. The user-defined gestures have been categorized into sets by the Piumsomboon [300]. This set can be utilized in AR applications for accomplishing different tasks. In addition, some of the mobile-based depth-sensing cameras are also under investigation. For instance, the SoftKinetic and Myo gesture armband controller. SodtKinetic is aimed at developing hand gesture interaction in mobile phones and wearable devices more accurately, while the Myo gesture armband controller is a biometric sensor that provides interaction in wearable and mobile environments.

### 8.5. Multimodal Interaction in AR

In addition to speech and gesture recognition, there are other types of voice recognition are being investigated. For example, the whistle-recognition system was developed by Lindeman [303] in mobile AR games. In this, the user had to whistle the right length and pitch to intimidate the virtual creatures in the game. Summary: The common input techniques and input methods have been examined in this section. These included simple information browsers and complex AR interfaces. The simple ones have very little support for the interaction and virtual content, while the complex interfaces were able to recognize even the speech and gesture inputs. A wide range of input methods are available for the AR interface; however, they are needed to be designed carefully. The following section delineates the research into the interface pattern, design, and guideline for AR experiences.

## 9. Design Guidelines and Interface Pattern

The previous section detailed the wide range of different AR input and interaction technologies; however, more rigorous research is required to design the AR experience. This section explores the interface patterns and design guidelines to develop an AR experience. The development of new interfaces goes through four main steps. First, the prototype is demonstrated. Second, interaction techniques are adopted from the other interface metaphors. Third, new interface metaphors are developed that are appropriate to the medium. Finally, the formal theoretical models are developed for modeling the interaction of users. In this regard, Wang et al. [304] employ user-centered AR instruction (UcAI) in procedural tasks. Thirty participants were selected for the experiment while having both the control and experiment groups. The result of the experiment suggested that introduction of UcAI increased the user’s spatial cognitive ability, particularly in the high-precision operational task. This research has the potential of guiding advanced AR instruction designs to perform tasks of high cognitive complexity. For instance, WIMP (windows, icons, menus, and pointers) is a very well-known desktop metaphor. In development, it has gone through all of these stages. There are methods developed that are used to predict the time taken by the mouse will select an icon of a given size. These are known as formal theoretical models. Fitts law [305] is among those models that help in determining the pointing times in the user interfaces. There are also a number of virtual reality interfaces available that are at the third stage with reference to the techniques available. For example, the manipulation and selection in immersive virtual worlds can be done by using the go-go interaction method [306]. On the other hand, as evident in the previous section, AR interfaces have barely surpassed the first two stages. Similarly, a number of AR interaction methods and technologies are available; however, by and large, they are only the extensions or versions of the existing 3D and 2D techniques present in mobiles, laptops, or AR interfaces. For instance, mobile phone experiences such as the gesture application and the touch screen input are added to AR. Therefore, there is a dire need to develop AR-specific interaction techniques and interface metaphors [307]. A deeper analysis and study of AR interfaces will help in the development of the appropriate metaphor interfaces. AR interfaces are unique in the sense that they need to develop close interaction between the real and the virtual worlds. A researcher, MacIntyre, has argued that the definition and the fusion of the virtual and real worlds are required for creating an AR design [308]. The primary goal of this is to depict the physical objects and user input onto the computer-generated graphics. This is done by using a suitable interaction interface. As a result, an AR design should have three components:

- The physical object.
- The virtual image to be developed.
- An interface to create an interaction between the physical world and the virtual objects.

Use of *design patterns* could be an alternative technique to develop the AR interface design. These design patterns are most commonly used in the fields of computer science and design interface. Alexander has defined the use of design patterns in the following words: “Each pattern describes a problem that occurs over and over again in our environment, and then describes the core of the solution to that problem in such a way that you can use this solution a million times over, without ever doing it the same way twice” [309,310]. The pattern language approach could be used to enhance AR development, as suggested by Reicher [311]. This idea has evolved from the earlier research works of MacWilliam [312]. This approach has two main functionalities. First, it is more focused on the software engineering aspect. Secondly, it suggests ways to develop complex AR systems by combining different modules of design patterns. So, they describe each pattern by the number of its aspects such as name, motivation, goal, description, consequences, known project usage, and general usability. One of the most notable examples of it is the DWARF framework [313]. DWARF is a component-based AR framework that is developed through the design pattern approach. In contrast to the pattern language approach, the user experience of design in the AR handheld device could be used for developing designs. This was described by Xu and the main concern was pre-patterns. Pre-patterns are the components that bridge the gap between the game design and the interaction design. For determining the method of using of design patterns, seamful design could be used. This suggests that the designer should integrate the AR handheld game design and the technology in such a way that they should blend into each other. Some users need more attention for designing effective AR experiences; therefore, the designing of special needs is another intervention to resolve this discrepancy. For instance, as pointed out by Rand and Maclntyre [314], in designing an AR system for the age group of 6–9, the developmental stages of the children should be accounted for in it. The research has also suggested that a powerful educational experience could be created through the use of AR. In addition to this development, it was also stated that the developmental stages of the students should be considered [315,316]. However, there is no extensive research that suggests the development of AR experiences for children [317]. Radu, in his paper, has determined the key four areas that should be considered while designing AR for children: attention, motor, special, logic, and memory abilities [318].

## 10. Security, Trust, and Collaborative AR

Security is very important in augmented reality, especially in collaborative augmented reality. While using collaborative AR applications, the data are exposed to external attacks, which increases concerns about security relating to AR technologies. Moreover, if the users who share the same virtual collaborative environments are unknown to each other, it also elevates these issues. In [319], the basic premise of the research is that the developed abstraction device not only improves the privacy but also the performance of the AR apps, which lays the groundwork for the development of future OS support for AR apps. The results suggested that the prototype enables secure offloading of heavyweight, incurs negligible overhead, and improves the overall performance of the app. In [320], the authors aim to resolve security and privacy challenges in multi-user AR applications. They have introduced an AR-sharing module along with systematized designs and representative case studies for functionality and security. This module is implemented as a prototype known as ArShare for the HoloLens. Finally, it also lays the foundation for the development of fully fledged and secure multi-user AR interaction. In [321], the authors used AR smart glasses to detail the “security and safety” aspect of AR applications as a case study. In the experiment, cloud-based architecture is linked to the oil extractor in combination with Vuzix Blade smart glasses. For security purposes, this app sends real-time signals if a dangerous situation arrives. In [322], deep learning is used to make the adaptive policies for generating the visual output in AR devices. Simulations are used that automatically detect the situation and generate policies and protect the system against disastrous malicious content. In [323], the authors discussed the case study of challenges faced by VR and AR in the field of security and privacy. The results showed that the attack reached the target of distance 1.5 m with 90 percent accuracy when using a four-digit password. In [324], the authors provide details and goals for developing security. They discuss the challenges faced in the development of edge computing architecture which also includes the discussion regarding reducing security risks. The main idea of the paper is to detail the design of security measures for both AR and non-AR devices. In [325], the authors presented that the handling of multi-user outputs and handling of data are demonstrated are the two main obstacles in achieving security and privacy of AR devices. It further provides new opportunities that can significantly improve the security and privacy realm of AR. In [326], the authors introduce the authentication tool for ensuring security and privacy in AR environments. For these purposes, the graphical user password is fused with the AR environments. A doodle password is created by the touch-gesture-recognition on a mobile phone, and then doodles are matched in real-time size. Additionally, doodles are matched with the AR environment. In [327], the authors discussed the immersive nature of augmented reality engenders significant threats in the realm of security and privacy. They further explore the aspects of securing buggy AR output. In [328], the authors employ the case study of an Android app, “Google Translator”, to detect and avoid variant privacy leaks. In addition, this research proposes the foundational framework to detect unnecessary privacy leaks. In [329], the authors discuss the AR security-related issues on the web. The security related vulnerabilities are identified and then engineering guidelines are proposed to make AR implementation secure. In [330], the past ten years of research work of the author, starting from 2011, in the field of augmented reality is presented. The main idea of the paper is to figure out the potential problems and to predict the future for the next ten years. It also explains the systematization for future work and focuses on evaluating AR security research. In [331], the authors presented various AR-related security issues and identified managing the virtual content in the real space as a challenge in making AR spaces secure for single and multi-users. The authors in [332] believe that there is a dire need of cybersecurity risks in the AR world. The introduction of systemized and universal policy modules for the AR architecture is a viable solution for mitigating security risks in AR. In [333], the authors discuss the challenge of enabling the different AR apps to augment the user’s world experience simultaneously, pointing out the conflicts between the AR applications.

## 11. Summary

**Figure 11:**
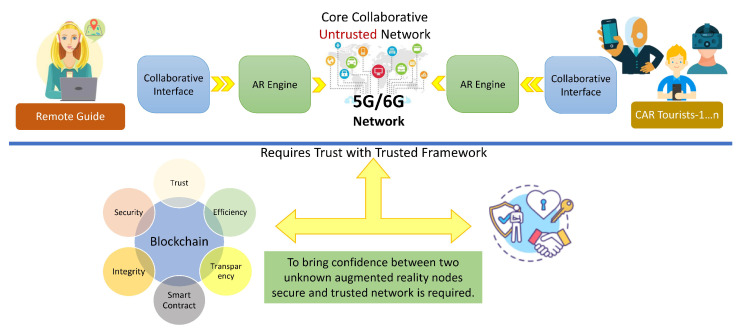
A model of blockchain-based trusted and secured collaborative AR system.

In this paper, the authors have reviewed the literature extensively in terms of tracking and displays technology, AR, and collaborative AR, as can be seen in Figure 10. It has been observed that collaborative AR has further two classifications i.e., co-located AR and remote collaboration [334]. Each of these collocated and remote collaborations has two further types i.e., synchronous and asynchronous. In remote collaborative AR, there are a number of use cases wherein it has been observed that *trust management* is too important a factor to consider because there are unknown parties that participate in remote activities to interact with each other and as such, they are unknown to each other as well [21,335,336,337,338]. There has been a lack of trust and security concerns during this remote collaboration. There are more chances of intrusion and vulnerabilities that can be possibly exploited [331,339,340]. One such collaboration is from the tourism sector, which has boosted the economy, especially in the pandemic era when physical interactors were not allowed [341]. To address these concerns, this research felt the need to ensure that the communication has integrity and for this purpose, the research utilized state-of-the-art blockchain infrastructure for collaborative applications in AR. The paper has proposed a complete secure framework wherein different applications working remotely are having a real feeling of trust in each other [17,342,343]. The participants within the collaborative AR subscribed to a trusted environment to further make interaction with each other in a secure fashion while their communication was protected through state-of-the-art blockchain infrastructure [338,344]. A model of such an application is shown in Figure 11.

**Figure 12:**
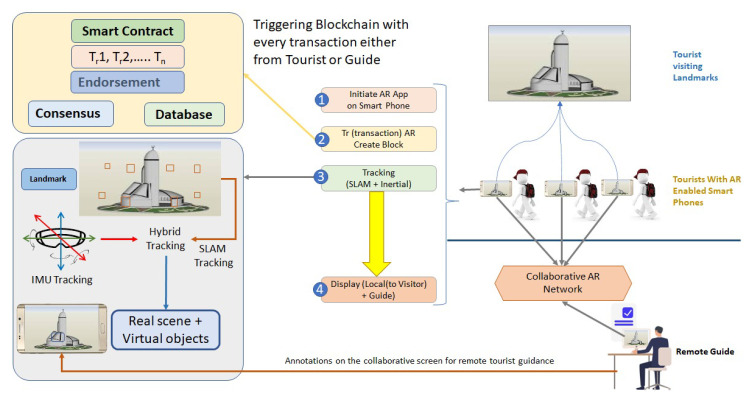
Sharing of the real-time environment of CAR tourist app for multiple users [16].

Figure 12 demonstrates the initiation of the AR App in step 1, while in step 2 of Figure 12, the blockchain is initiated to record transactions related to sign-up, record audio calls, proceed with payment/subscription, etc. In step 3, when the transaction is established, AR is initiated, which enables the visitor to receive guidance from the travel guide. The app creates a map of the real environment. The created map and the vision provide a SLAM, i.e., SLAM provides an overall vision and details of different objects in the real world. Inertial tracking controls the movement and direction in the augmented reality application. The virtual objects are then placed after identifying vision and tracking. In a collaborative environment, the guides are provided with an option of annotation so they can circle a particular object or spot different locations and landmarks or point to different incidents [16].

## 12. Directions for Research

The commercialization efforts of companies have made AR a mainstream field. However, for the technology to reach its full potential, the number of research areas should be expanded. Azuma has explained the three major obstacles in the way of AR: interface limitation, technological limitations, and the issue of social acceptance. In order to overcome these barriers, the two major models are developed: first, Roger’s innovation diffusion theory [345] and the technology acceptance model (developed by Martinez) [346]. Roger has explained the following major restriction towards the adoption of this technology: limited computational power of AR technology, social acceptance, no AR standards, tracking inaccuracy, and overloading of information. The main research trends in display technology, user interface, and tracking were identified by Zho by evaluating ten years of ISMAR papers. The research has been conducted in a wide number of areas except for social acceptance. This section aims at exploring future opportunities and ongoing research in the field of AR, particularly in the four key areas: display, tracking, interaction, and social acceptance. Moreover, there are a number of other topics including evaluation techniques, visualization methods, applications, authoring and content-creating tools, rendering methods, and some other areas.

## 13. Conclusions

This document has detailed a number of research papers that address certain problems of AR. For instance, AR tracking techniques are detailed in Section 3. Display technologies, such as VST and OST, and its related calibration techniques in Section 4, authoring tools in Section 6, collaborative AR in Section 7, AR interaction in Section 8, and design guidelines in Section 9. Finally, promising security and trust-related papers are discussed in the final section. We presented the problem statement and a short solution to the problem is provided. These aspects should be covered in future research and the most pertinent among these are the hybrid AR interfaces, social acceptance, etc. The speed of research is significantly increasing, and AR technology is going to dramatically impact our lives in the next 20 years.

## Figures and Tables

**Table 2:** A Summary of Augmented Reality Display Technologies.

No.	Type	Technology Is Still Used or Obselete?	Technology Used in Devices/Software/Company	How Does It Work?	Advantages	Challenges	Practical Use Areas	Example Studies
1	Optical See-through	Yes	i. Microsoft’s Hololens ii. Magic Leap One iii. Google Glass	Merges virtual and real scenes using optical systems through which users can see	+the real world can be viewed +achieves immersive augmented reality experiences	-system lags and calibration issues -reflections and limited field of view -occlusion may be challenging to achieve	Medicine Tourism Education	[11,102,112,113,114,140,141,142,143,144,145,146,147,148,149,150,151]
2	Video See-through	Yes	i. HTC Vive Headset ii. Handheld Devices with AR Library, such as, ARCore, ARKit	Combines a digital video of the physical world with virtual content using image processing	+enables a wide field of view +leveraging brightness of objects	-weak peripheral vision of the visuals -lags due to video rendering -disorientation	Advertisement Tourism	[156,157]
3	Projection based	Yes	Tile Five	Projects the virtual scene on a physical object (i.e., Wall or Ceiling) using a projector	+the user does not need to wear any equipment	-The projection is static -Projections are restricted to only one location	Entertainment	[158,159,160,161,162,163,164]
4	Eye multiplexed	Yes	Real Wear HMT-1	Integrates real scenes and virtual content in the mind of users	+requires less computational power	-Display must be close to the viewer’s eyes		[72]
5	Head attached	Yes	SketchUp	Displays virtual images in front of the users’ eyes using dedicatedequipment (e.g., helmets and glasses)	+does not block users’ vision +enables user immersion and engagement	-Intrusive to wear -user and environment tracking could be challenging	Architecture Training	[88,165,166,167,168]
6	Head mounted	Yes	i. Avionic Displays ii. Solos iii. Beyeonics	Shows AR experiences in front of the users’ eyes using HMDs	+VR world is compact in the smallest physical space +enables higher user focus on interaction with AR	-Must be worn, which could be disturbing -Lenses may impact the user experience	Education Medicine Healthcare	[208,209,210,211,212,213]
7	Body attached and handheld	Yes	Android iOS	Depicts AR visuals on regular handheld devices	+availability of affordable devices and apps +ubiquitous devices (e.g. smartphones) +ability to work with haptic and audio sensors	-interaction on tangible devices poses difficulty -visibility of handheld devices (e.g., brightness and contract)	Leisure	[177,178,179,180,181,182]

**Table 3:** A summary of development and authoring tools for augmented reality application development.

Authoring Tool	AR Component	Features	Research Based or Commercial	Active/Not	Used in/by Software/Tool	Platform Supported
OpenScene	Graph Library	-OpenScene is a graph library -Can be linked with OpenGL and osgART	Researched/Commercial	Active	ARToolKit	GNULinux/Windows/OSX
PTAM	SLAM Tracking Library	OpenSource/Available Under GPL	Research-Based	Can be used for research and open source. However, for productionARCore/ARKit implementation of SLAMis available/Not Active	Standalone	Linux/OSX
BazAR	Tracking and Geometric Calibration	OpenSource/Available Under GPL	Research-Based	Can beused for research to detectan object via camera, calibrate it and initiatetracking to put a basicvirtual image onit/Not Active	Standalone	Linux/Windows
Goblin XNA	-Platform for Mobile-based AR -Marker Based tracking with ARTag	Free Windows Platform	Research/Education Based	Can beused forresearch and educationpurposes, to generate 3Dand track the object/NotActive	Standalone	Windows
Studierstube	Open Tracker	-Open Source/Free -Have Builtin Hardware Tracking -Used for Collaborative AR	Research/Education Based	Can beused forresearch and educationpurposes to test varioustracking and AR apps/NotActive	Standalone	Linux
Metaio SDK	Image, Marker, Face, infrared, and 3D object Tracking	-Support Localization -Tracking	The source code can be provided after proper owner’s approval on their website	Active	Standalone	Andoird/iOS
ARTag	-Maker-Based (Fiducial) Tracking	Tracking Library that support AR application development	No support available	Not Active	Standalone	Windows
WikiTude Studio	-SLAM -Image Tracking -Calibration Manager -Geo AR -Inertial	It is an SDK that can help to build an AR app without any other tools needed for Android, iOS, Windows, and Linux.	Commercial	Active	Native API, JavaScript API, Unity Plugin, Cordova Plugin, Flutter Plugin,	Windows, Linux, iOS, Android
BuildAR	Marker based tracking	-Standalone easy to create new AR applications. -	Free	Not Active	Standalone	Windows
AMIRE and CATOMIR	Standalone ARTools		No support availble	Active		
ARCore	SLAM + Inertial forTracking and understanding the environment Integrated Display	ARCore support Motion tracking with SLAM and Inertial, Depth Understanding, Light Estemation,	Free	Active	Android, Android NDK, Unity(AR Foundation), iOS, Unreal, Web	Android, iOS
MS HoloLense	-Vision Based Tracking -OST Display -VST Display	Is an augmented reality headset for running AR apps	Commercial	Active	Unity, Unreal, Vuforia	Windows 10
ARKit	-Motion Tracking -Camera Scene Capture -Advanced Scene Processing	With ARKit one can create a complete AR application. It has tracking, display and development environment to develop AR app.	Commercial for application development	Active	Plugin Available for Unity	iOS
Vuforia	Supports -Marker less (vision-based) and -Marker based tracking (Fiducial) -Calibration Library	-A complete SDK for AR application development. -Supports many languages for AR development for API - C++, Java, Net	Free and Commercial both versions are available.	Active	-Standalone Native development -Plugin available for Unity	iOS, Android
ARToolKit	-Tracking Library Supports both -Video See Through(VST) -Optical See Through(OST) -ARTag variant of ARToolKit supports Marker based(Fiducial)	-C and C++ Language Support for AR -JARToolKit for Java Support -A Modified Marker Base -ARToolKitPlus	Free and Commercial both are available.	Active	-Standalone -Unity plugin is also available for Integration with Unity libraries	Linux, Windows, McOS X
DeepAR (Creator Studio)	Embdded Tracking, VST Display	Standalone easy to create AR applications for non-programmers	Commercial	Active	DeepAR SDK Web SDK	Windows, iOS, Android,

## References

[1] Berciu A.G., Dulf E.H., Stefan I.A. (2022). Flexible Augmented Reality-Based Health Solution for Medication Weight Establishment. Processes.

[2] Sırakaya M., Alsancak Sırakaya D. (2022). Augmented reality in STEM education: A systematic review. Interact. Learn. Environ..

[3] Chouchene A., Ventura Carvalho A., Charrua-Santos F., Barhoumi W. (2022). Augmented Reality-Based Framework Supporting Visual Inspection for Automotive Industry. Appl. Syst. Innov..

[4] (2022). Augmented Reality and Virtual Reality Market. https://www.marketsandmarkets.com/Market-Reports/augmented-reality-virtual-reality-market-1185.html?gclid=CjwKCAjwtKmaBhBMEiwAyINuwNi_hv87XEg2rZqpBVQGtOr1gL8mNrLbRsYaSZmNYOXF4Za63Bhb4xoChZkQAvD_BwE.

[5] Lee W.H., Lee H.K. (2016). The usability attributes and evaluation measurements of mobile media AR (augmented reality). Cogent Arts Humanit..

[6] Mourtzis D., Angelopoulos J., Panopoulos N. (2022). Challenges and Opportunities for Integrating Augmented Reality and Computational Fluid Dynamics Modeling under the Framework of Industry 4.0. Procedia CIRP.

[7] Carter D. (2022). Immersive Employee Experiences in the Metaverse: Virtual Work Environments, Augmented Analytics Tools, and Sensory and Tracking Technologies. Psychosociological Issues Hum. Resour. Manag..

[8] Zhang Z., Wen F., Sun Z., Guo X., He T., Lee C. (2022). Artificial Intelligence-Enabled Sensing Technologies in the 5G/Internet of Things Era: From Virtual Reality/Augmented Reality to the Digital Twin. Adv. Intell. Syst..

[9] Yu J., Denham A.R., Searight E. (2022). A systematic review of augmented reality game-based Learning in STEM education. Educ. Technol. Res. Dev..

[10] Moro M., Marchesi G., Hesse F., Odone F., Casadio M. (2022). Markerless vs. Marker-Based Gait Analysis: A Proof of Concept Study. Sensors.

[11] Pulli K. (2017). 11–2: Invited Paper: Meta 2: Immersive Optical-See-Through Augmented Reality. SID Symposium Digest of Technical Papers.

[12] Wilson A., Hua H. (2019). Design and demonstration of a vari-focal optical see-through head-mounted display using freeform Alvarez lenses. Opt. Express.

[13] Khan D., Ullah S., Yan D.M., Rabbi I., Richard P., Hoang T., Billinghurst M., Zhang X. (2018). Robust tracking through the design of high quality fiducial markers: An optimization tool for ARToolKit. IEEE Access.

[14] Ramadar P. (2014). NS Flartoolkit Flash Augmented Reality Alt Actionscript, Journal=Buku AR Online Solo. https://artoolworks.com/products/open-source-software/flartoolkit-2.html.

[15] Linowes J., Babilinski K. (2017). Augmented Reality for Developers: Build Practical Augmented Reality Applications with Unity, ARCore, ARKit, and Vuforia.

[16] Syed T.A., Jan S., Siddiqui M.S., Alzahrani A., Nadeem A., Ali A., Ullah A. (2022). CAR-Tourist: An Integrity-Preserved Collaborative Augmented Reality Framework-Tourism as a Use-Case. Appl. Sci..

[17] Syed T.A., Alzahrani A., Jan S., Siddiqui M.S., Nadeem A., Alghamdi T. (2019). A comparative analysis of blockchain architecture and its applications: Problems and recommendations. IEEE Access.

[18] Agati S.S., Bauer R.D., Hounsell M.d.S., Paterno A.S. Augmented reality for manual assembly in industry 4.0: Gathering guidelines. Proceedings of the 2020 22nd Symposium on Virtual and Augmented Reality (SVR).

[19] Brooker J. (2007). The polytechnic ghost: Pepper’s ghost, metempsychosis and the magic lantern at the Royal Polytechnic Institution. Early Pop. Vis. Cult..

[20] Drascic D., Grodski J.J., Milgram P., Ruffo K., Wong P., Zhai S. ARGOS: A display system for augmenting reality. Proceedings of the INTERACT’93 and CHI’93 Conference on Human Factors in Computing Systems.

[21] Abdeen M., Jan S., Khan S., Ali T. (2019). Employing Takaful Islamic Banking Through State of The Art Blockchain: A Case Study. Int. J. Adv. Comput. Sci. Appl..

[22] Kerber R. (2008). Advanced tactic targeted grocer. The Boston Globe.

[23] Mansfield-Devine S. (2012). Interview: BYOD and the enterprise network. Comput. Fraud Secur..

[24] Nofer M., Gomber P., Hinz O., Schiereck D. (2017). Blockchain. Bus. Inf. Syst. Eng..

[25] Behzadan A.H., Aziz Z., Anumba C.J., Kamat V.R. (2008). Ubiquitous location tracking for context-specific information delivery on construction sites. Autom. Constr..

[26] Bottani E., Vignali G. (2019). Augmented reality technology in the manufacturing industry: A review of the last decade. IISE Trans..

[27] Sereno M., Wang X., Besançon L., McGuffin M.J., Isenberg T. (2020). Collaborative work in augmented reality: A survey. IEEE Trans. Vis. Comput. Graph..

[28] Ens B., Quigley A., Yeo H.S., Irani P., Piumsomboon T., Billinghurst M. Counterpoint: Exploring mixed-scale gesture interaction for AR applications. Proceedings of the Extended Abstracts of the 2018 CHI Conference on Human Factors in Computing Systems.

[29] Hettig J., Engelhardt S., Hansen C., Mistelbauer G. (2018). AR in VR: Assessing surgical augmented reality visualizations in a steerable virtual reality environment. Int. J. Comput. Assist. Radiol. Surg..

[30] Goh E.S., Sunar M.S., Ismail A.W. (2019). 3D object manipulation techniques in handheld mobile augmented reality interface: A review. IEEE Access.

[31] Kollatsch C., Klimant P. (2021). Efficient integration process of production data into Augmented Reality based maintenance of machine tools. Prod. Eng..

[32] Bhattacharyya P., Nath R., Jo Y., Jadhav K., Hammer J. Brick: Toward a model for designing synchronous colocated augmented reality games. Proceedings of the 2019 CHI Conference on Human Factors in Computing Systems.

[33] Kim K., Billinghurst M., Bruder G., Duh H.B.L., Welch G.F. (2018). Revisiting trends in augmented reality research: A review of the 2nd decade of ISMAR (2008–2017). IEEE Trans. Vis. Comput. Graph..

[34] Liu H., Wang L. (2017). An AR-based worker support system for human-robot collaboration. Procedia Manuf..

[35] Cortés-Dávalos A., Mendoza S. Collaborative Web Authoring of 3D Surfaces Using Augmented Reality on Mobile Devices. Proceedings of the 2016 IEEE/WIC/ACM International Conference on Web Intelligence (WI).

[36] Qiao X., Ren P., Dustdar S., Liu L., Ma H., Chen J. (2019). Web AR: A promising future for mobile augmented reality—State of the art, challenges, and insights. Proc. IEEE.

[37] Shukri S.A.A., Arshad H., Abidin R.Z. (2017). The design guidelines of mobile augmented reality for tourism in Malaysia. AIP Conference Proceedings.

[38] Fallahkhair S., Brito C.A. (2019). Design Guidelines for Development of Augmented Reality Application with Mobile and Wearable Technologies for Contextual Learning. Braz. J. Technol. Commun. Cogn. Sci..

[39] Akçayır M., Akçayır G. (2017). Advantages and challenges associated with augmented reality for education: A systematic review of the literature. Educ. Res. Rev..

[40] da Silva M.M., Teixeira J.M.X., Cavalcante P.S., Teichrieb V. (2019). Perspectives on how to evaluate augmented reality technology tools for education: A systematic review. J. Braz. Comput. Soc..

[41] Sarkar P., Pillai J.S., Gupta A. ScholAR: A collaborative learning experience for rural schools using Augmented Reality application. Proceedings of the 2018 IEEE Tenth International Conference on Technology for Education (T4E).

[42] Soleimani H., Jalilifar A., Rouhi A., Rahmanian M. (2019). Augmented Reality and Virtual Reality Scaffoldings in Improving the Abstract Genre Structure in a Collaborative Learning Environment: A CALL Study. J. Engl. Lang. Teach. Learn..

[43] Hadar E. (2018). Toward Development Tools for Augmented Reality Applications—A Practitioner Perspective. Workshop on Enterprise and Organizational Modeling and Simulation.

[44] Mukhametshin S., Makhmutova A., Anikin I. Sensor tag detection, tracking and recognition for AR application. Proceedings of the 2019 International Conference on Industrial Engineering, Applications and Manufacturing (ICIEAM).

[45] Santoni F., De Angelis A., Moschitta A., Carbone P. (2021). MagIK: A Hand-Tracking Magnetic Positioning System Based on a Kinematic Model of the Hand. IEEE Trans. Instrum. Meas..

[46] Frikha R., Ejbali R., Zaied M. Handling occlusion in augmented reality surgical training based instrument tracking. Proceedings of the 2016 IEEE/ACS 13th International Conference of Computer Systems and Applications (AICCSA).

[47] Wang M., Shi Q., Song S., Meng M.Q.H. (2020). A novel magnetic tracking approach for intrabody objects. IEEE Sensors J..

[48] Davis F.D., Bagozzi R.P., Warshaw P.R. (1989). User acceptance of computer technology: A comparison of two theoretical models. Manag. Sci..

[49] Davison A.J., Reid I.D., Molton N.D., Stasse O. (2007). MonoSLAM: Real-time single camera SLAM. IEEE Trans. Pattern Anal. Mach. Intell..

[50] De Smet J. (2014). The Smart Contact Lens: From an Artificial Iris to a Contact Lens Display. Ph.D. Thesis.

[51] Dissanayake M.G., Newman P., Clark S., Durrant-Whyte H.F., Csorba M. (2001). A solution to the simultaneous localization and map building (SLAM) problem. IEEE Trans. Robot. Autom..

[52] Dodgson N.A. (2005). Autostereoscopic 3D displays. Computer.

[53] De Smet J., Avci A., Joshi P., Schaubroeck D., Cuypers D., De Smet H. (2013). Progress toward a liquid crystal contact lens display. J. Soc. Inf. Disp..

[54] Heidemann G., Bax I., Bekel H. Multimodal interaction in an augmented reality scenario. Proceedings of the 6th International Conference on Multimodal Interfaces.

[55] Chakrabarty A., Morris R., Bouyssounouse X., Hunt R. Autonomous indoor object tracking with the Parrot AR. Drone. Proceedings of the 2016 International Conference on Unmanned Aircraft Systems (ICUAS).

[56] Buttner S., Sand O., Rocker C. (2017). Exploring design opportunities for intelligent worker assistance: A new approach using projetion-based AR and a novel hand-tracking algorithm. Proceedings of the European Conference on Ambient Intelligence.

[57] Gupta S., Chaudhary R., Gupta S., Kaur A., Mantri A. A survey on tracking techniques in augmented reality based application. Proceedings of the 2019 Fifth International Conference on Image Information Processing (ICIIP).

[58] Krishna V., Ding Y., Xu A., Höllerer T. Multimodal biometric authentication for VR/AR using EEG and eye tracking. Proceedings of the Adjunct of the 2019 International Conference on Multimodal Interaction.

[59] Dzsotjan D., Ludwig-Petsch K., Mukhametov S., Ishimaru S., Kuechemann S., Kuhn J. The Predictive Power of Eye-Tracking Data in an Interactive AR Learning Environment. Proceedings of the Adjunct Proceedings of the 2021 ACM International Joint Conference on Pervasive and Ubiquitous Computing and Proceedings of the 2021 ACM International Symposium on Wearable Computers.

[60] Chen L., Cao C., De la Torre F., Saragih J., Xu C., Sheikh Y. High-fidelity Face Tracking for AR/VR via Deep Lighting Adaptation. Proceedings of the IEEE/CVF Conference on Computer Vision and Pattern Recognition.

[61] Rambach J., Pagani A., Schneider M., Artemenko O., Stricker D. (2018). 6DoF object tracking based on 3D scans for augmented reality remote live support. Computers.

[62] Ha T., Billinghurst M., Woo W. (2012). An interactive 3D movement path manipulation method in an augmented reality environment. Interact. Comput..

[63] Syahidi A.A., Tolle H., Supianto A.A., Arai K. AR-Child: Analysis, Evaluation, and Effect of Using Augmented Reality as a Learning Media for Preschool Children. Proceedings of the 2019 5th International Conference on Computing Engineering and Design (ICCED).

[64] Wang X., Dunston P.S. (2008). User perspectives on mixed reality tabletop visualization for face-to-face collaborative design review. Autom. Constr..

[65] Wang X., Dunston P.S. (2011). Comparative effectiveness of mixed reality-based virtual environments in collaborative design. IEEE Trans. Syst. Man Cybern. Part C.

[66] Hauptmann A.G. Speech and gestures for graphic image manipulation. Proceedings of the SIGCHI Conference on Human Factors in Computing Systems.

[67] Heath C., Luff P. Disembodied conduct: Communication through video in a multi-media office environment. Proceedings of the SIGCHI Conference on Human Factors in Computing Systems.

[68] Rambach J., Pagani A., Stricker D. [poster] Augmented things: Enhancing AR applications leveraging the internet of things and universal 3D object tracking. Proceedings of the 2017 IEEE International Symposium on Mixed and Augmented Reality (ISMAR-Adjunct).

[69] Viyanon W., Songsuittipong T., Piyapaisarn P., Sudchid S. AR furniture: Integrating augmented reality technology to enhance interior design using marker and markerless tracking. Proceedings of the 2nd International Conference on Intelligent Information Processing.

[70] Turkan Y., Radkowski R., Karabulut-Ilgu A., Behzadan A.H., Chen A. (2017). Mobile augmented reality for teaching structural analysis. Adv. Eng. Inform..

[71] Dorfmüller K. (1999). Robust tracking for augmented reality using retroreflective markers. Comput. Graph..

[72] Danielsson O., Holm M., Syberfeldt A. (2020). Augmented reality smart glasses for operators in production: Survey of relevant categories for supporting operators. Procedia CIRP.

[73] Dörner R., Geiger C., Haller M., Paelke V. (2003). Authoring mixed reality—A component and framework-based approach. Entertainment Computing.

[74] Drascic D., Milgram P. Positioning accuracy of a virtual stereographic pointer in a real stereoscopic video world. Proceedings of the Stereoscopic Displays and Applications II.

[75] Drascic D., Milgram P. Perceptual issues in augmented reality. Proceedings of the Stereoscopic Displays and Virtual Reality Systems III. International Society for Optics and Photonics.

[76] Dünser A. (2008). Supporting low ability readers with interactive augmented reality. Annu. Rev. Cybertherapy Telemed..

[77] Dünser A., Hornecker E. Lessons from an AR book study. Proceedings of the 1st International Conference on Tangible and Embedded Interaction.

[78] Gibson L., Hanson V.L. Digital motherhood: How does technology help new mothers?. Proceedings of the SIGCHI Conference on Human Factors in Computing Systems.

[79] Wuest H., Engekle T., Wientapper F., Schmitt F., Keil J. From CAD to 3D Tracking—Enhancing & Scaling Model-Based Tracking for Industrial Appliances. Proceedings of the 2016 IEEE International Symposium on Mixed and Augmented Reality (ISMAR-Adjunct).

[80] LaViola J.J. (2000). A discussion of cybersickness in virtual environments. ACM SIGCHI Bull..

[81] Gao Y.F., Wang H.Y., Bian X.N. Marker tracking for video-based augmented reality. Proceedings of the 2016 International Conference on Machine Learning and Cybernetics (ICMLC).

[82] Szalavári Z., Eckstein E., Gervautz M. Collaborative gaming in augmented reality. Proceedings of the ACM Symposium on Virtual Reality Software and Technology.

[83] Dolata M., Agotai D., Schubiger S., Schwabe G. (2019). Pen-and-paper rituals in service interaction: Combining high-touch and high-tech in financial advisory encounters. Proc. ACM Hum.-Comput. Interact..

[84] Butz A., Hollerer T., Feiner S., MacIntyre B., Beshers C. Enveloping users and computers in a collaborative 3D augmented reality. Proceedings of the 2nd IEEE and ACM International Workshop on Augmented Reality (IWAR’99).

[85] Müller J., Rädle R., Reiterer H. Remote collaboration with mixed reality displays: How shared virtual landmarks facilitate spatial referencing. Proceedings of the 2017 CHI Conference on Human Factors in Computing Systems.

[86] Gül L.F. (2018). Studying gesture-based interaction on a mobile augmented reality application for co-design activity. J. Multimodal User Interfaces.

[87] Benko H., Ishak E.W., Feiner S. Collaborative mixed reality visualization of an archaeological excavation. Proceedings of the Third IEEE and ACM International Symposium on Mixed and Augmented Reality.

[88] Franz J., Alnusayri M., Malloch J., Reilly D. (2019). A comparative evaluation of techniques for sharing AR experiences in museums. Proc. ACM Hum.-Comput. Interact..

[89] An Z., Xu X., Yang J., Liu Y., Yan Y. (2018). Research of the three-dimensional tracking and registration method based on multiobjective constraints in an AR system. Appl. Opt..

[90] Oskiper T., Samarasekera S., Kumar R. [POSTER] CamSLAM: Vision Aided Inertial Tracking and Mapping Framework for Large Scale AR Applications. Proceedings of the 2017 IEEE International Symposium on Mixed and Augmented Reality (ISMAR-Adjunct).

[91] Zhang W., Han B., Hui P. Jaguar: Low latency mobile augmented reality with flexible tracking. Proceedings of the 26th ACM International Conference on Multimedia.

[92] Tokusho Y., Feiner S. (2009). Prototyping an outdoor mobile augmented reality street view application. ISMAR Workshop on Outdoor Mixed and Augmented Reality.

[93] Henrysson A., Ollila M. UMAR: Ubiquitous mobile augmented reality. Proceedings of the 3rd International Conference on Mobile and Ubiquitous Multimedia.

[94] Henrysson A., Ollila M., Billinghurst M. Mobile phone based AR scene assembly. Proceedings of the 4th International Conference on Mobile and Ubiquitous Multimedia.

[95] Hilliges O., Kim D., Izadi S., Weiss M., Wilson A. HoloDesk: Direct 3d interactions with a situated see-through display. Proceedings of the SIGCHI Conference on Human Factors in Computing Systems.

[96] Ar Y., Ünal M., Sert S.Y., Bostanci E., Kanwal N., Güzel M.S. Evolutionary Fuzzy Adaptive Motion Models for User Tracking in Augmented Reality Applications. Proceedings of the 2018 2nd International Symposium on Multidisciplinary Studies and Innovative Technologies (ISMSIT).

[97] Ashutosh K. Hardware Performance Analysis of Mobile-Based Augmented Reality Systems. Proceedings of the 2020 International Conference on Computational Performance Evaluation (ComPE).

[98] Yang X., Fan X., Wang J., Yin X., Qiu S. (2020). Edge-based cover recognition and tracking method for an AR-aided aircraft inspection system. Int. J. Adv. Manuf. Technol..

[99] Kang D., Heo J., Kang B., Nam D. (2019). Pupil detection and tracking for AR 3D under various circumstances. Electron. Imaging.

[100] Hu X., Hua H. (2012). Design of an optical see-through multi-focal-plane stereoscopic 3d display using freeform prisms. Frontiers in Optics.

[101] Hourcade J.P. (2008). Interaction Design and Children.

[102] Kang D., Ma L. (2021). Real-Time Eye Tracking for Bare and Sunglasses-Wearing Faces for Augmented Reality 3D Head-Up Displays. IEEE Access.

[103] Jeong J., Lee C.K., Lee B., Lee S., Moon S., Sung G., Lee H.S., Lee B. (2020). Holographically printed freeform mirror array for augmented reality near-eye display. IEEE Photonics Technol. Lett..

[104] Park S.g. (2021). Augmented and mixed reality optical see-through combiners based on plastic optics. Inf. Disp..

[105] Lee Y.H., Zhan T., Wu S.T. (2019). Prospects and challenges in augmented reality displays. Virtual Real. Intell. Hardw..

[106] Jang C., Mercier O., Bang K., Li G., Zhao Y., Lanman D. (2020). Design and fabrication of freeform holographic optical elements. ACM Trans. Graph. (TOG).

[107] Yu C., Peng Y., Zhao Q., Li H., Liu X. (2017). Highly efficient waveguide display with space-variant volume holographic gratings. Appl. Opt..

[108] Gorovyi I.M., Sharapov D.S. Advanced image tracking approach for augmented reality applications. Proceedings of the 2017 Signal Processing Symposium (SPSympo).

[109] Hix D., Gabbard J.L., Swan J.E., Livingston M.A., Hollerer T.H., Julier S.J., Baillot Y., Brown D. A cost-effective usability evaluation progression for novel interactive systems. Proceedings of the 37th Annual Hawaii International Conference on System Sciences.

[110] Hodges S., Williams L., Berry E., Izadi S., Srinivasan J., Butler A., Smyth G., Kapur N., Wood K. (2006). SenseCam: A retrospective memory aid. Proceedings of the International Conference on Ubiquitous Computing.

[111] Isham M.I.M., Mohamed F., Siang C.V., Yusoff Y.A., Abd Aziz A.A., Dewi D.E.O. A framework of ultrasounds image slice positioning and orientation in 3D augmented reality environment using hybrid tracking method. Proceedings of the 2018 IEEE Conference on Big Data and Analytics (ICBDA).

[112] Park J.H., Kim S.B. (2018). Optical see-through holographic near-eye-display with eyebox steering and depth of field control. Opt. Express.

[113] Chakravarthula P., Peng Y., Kollin J., Fuchs H., Heide F. (2019). Wirtinger holography for near-eye displays. ACM Trans. Graph. (TOG).

[114] Peng Y., Choi S., Padmanaban N., Wetzstein G. (2020). Neural holography with camera-in-the-loop training. ACM Trans. Graph. (TOG).

[115] Ruan W., Yao L., Sheng Q.Z., Falkner N.J., Li X. Tagtrack: Device-free localization and tracking using passive rfid tags. Proceedings of the 11th International Conference on Mobile and Ubiquitous Systems: Computing, Networking and Services.

[116] Ellis S.R., Menges B.M. (2001). Studies of the Localization of Virtual Objects in the Near Visual Field. Fundamentals of Wearable Computers and Augmented Reality.

[117] Evennou F., Marx F. (2006). Advanced integration of WiFi and inertial navigation systems for indoor mobile positioning. EURASIP J. Adv. Signal Process..

[118] Bach B., Sicat R., Pfister H., Quigley A. (2017). Drawing into the AR-CANVAS: Designing embedded visualizations for augmented reality. Workshop on Immersive Analytics.

[119] Zeng H., He X., Pan H. (2019). FunPianoAR: A novel AR application for piano learning considering paired play based on multi-marker tracking. J. Phys. Conf. Ser..

[120] Rewkowski N., State A., Fuchs H. Small Marker Tracking with Low-Cost, Unsynchronized, Movable Consumer Cameras For Augmented Reality Surgical Training. Proceedings of the 2020 IEEE International Symposium on Mixed and Augmented Reality Adjunct (ISMAR-Adjunct).

[121] Hoffman H.G. Physically touching virtual objects using tactile augmentation enhances the realism of virtual environments. Proceedings of the IEEE 1998 Virtual Reality Annual International Symposium (Cat. No. 98CB36180).

[122] Mao W., He J., Qiu L. Cat: High-precision acoustic motion tracking. Proceedings of the 22nd Annual International Conference on Mobile Computing and Networking.

[123] Höllerer T., Wither J., DiVerdi S. (2007). “Anywhere augmentation”: Towards mobile augmented reality in unprepared environments. Location Based Services and TeleCartography.

[124] Hong J. (2013). Considering privacy issues in the context of Google glass. Commun. ACM.

[125] Hua H., Brown L.D., Gao C., Ahuja N. (2003). A new collaborative infrastructure: SCAPE. Proceedings of the IEEE Virtual Reality, 2003. Proceedings.

[126] Huang Y., Weng D., Liu Y., Wang Y. Key issues of wide-area tracking system for multi-user augmented reality adventure game. Proceedings of the 2009 Fifth International Conference on Image and Graphics.

[127] Huber M., Pustka D., Keitler P., Echtler F., Klinker G. A system architecture for ubiquitous tracking environments. Proceedings of the 2007 6th IEEE and ACM International Symposium on Mixed and Augmented Reality.

[128] Hugues O., Fuchs P., Nannipieri O. (2011). New augmented reality taxonomy: Technologies and features of augmented environment. Handbook of Augmented Reality.

[129] Inami M., Kawakami N., Sekiguchi D., Yanagida Y., Maeda T., Tachi S. (2000). Visuo-haptic display using head-mounted projector. Proceedings of the Proceedings IEEE Virtual Reality 2000 (Cat. No. 00CB37048).

[130] Ekman P., Friesen W.V. (2010). The Repertoire of Nonverbal Behavior: Categories, Origins, Usage, and Coding.

[131] Ellis S.R., Menges B.M. (1997). Judgments of the distance to nearby virtual objects: Interaction of viewing conditions and accommodative demand. Presence Teleoperators Virtual Environ..

[132] Grubert J., Itoh Y., Moser K., Swan J.E. (2017). A survey of calibration methods for optical see-through head-mounted displays. IEEE Trans. Vis. Comput. Graph..

[133] Moser K., Itoh Y., Oshima K., Swan J.E., Klinker G., Sandor C. (2015). Subjective evaluation of a semi-automatic optical see-through head-mounted display calibration technique. IEEE Trans. Vis. Comput. Graph..

[134] Ballestin G., Chessa M., Solari F. (2021). A registration framework for the comparison of video and optical see-through devices in interactive augmented reality. IEEE Access.

[135] Cattari N., Cutolo F., D’amato R., Fontana U., Ferrari V. (2019). Toed-in vs parallel displays in video see-through head-mounted displays for close-up view. IEEE Access.

[136] Banks M.S., Read J.C., Allison R.S., Watt S.J. (2012). Stereoscopy and the human visual system. SMPTE Motion Imaging J..

[137] Cutolo F., Fontana U., Ferrari V. (2018). Perspective preserving solution for quasi-orthoscopic video see-through HMDs. Technologies.

[138] State A., Keller K.P., Fuchs H. Simulation-based design and rapid prototyping of a parallax-free, orthoscopic video see-through head-mounted display. Proceedings of the Fourth IEEE and ACM International Symposium on Mixed and Augmented Reality (ISMAR’05).

[139] Bottecchia S., Cieutat J.M., Merlo C., Jessel J.P. (2009). A new AR interaction paradigm for collaborative teleassistance system: The POA. Int. J. Interact. Des. Manuf. (IJIDeM).

[140] Xiong J., Hsiang E.L., He Z., Zhan T., Wu S.T. (2021). Augmented reality and virtual reality displays: Emerging technologies and future perspectives. Light. Sci. Appl..

[141] Do H., Kim Y.M., Min S.W. (2019). Focus-free head-mounted display based on Maxwellian view using retroreflector film. Appl. Opt..

[142] Song W., Cheng Q., Surman P., Liu Y., Zheng Y., Lin Z., Wang Y. (2019). Design of a light-field near-eye display using random pinholes. Opt. Express.

[143] Wang X., Hua H. (2021). Depth-enhanced head-mounted light field displays based on integral imaging. Opt. Lett..

[144] Huang H., Hua H. (2018). High-performance integral-imaging-based light field augmented reality display using freeform optics. Opt. Express.

[145] Kim S.B., Park J.H. (2018). Optical see-through Maxwellian near-to-eye display with an enlarged eyebox. Opt. Lett..

[146] Shrestha P.K., Pryn M.J., Jia J., Chen J.S., Fructuoso H.N., Boev A., Zhang Q., Chu D. (2019). Accommodation-free head mounted display with comfortable 3D perception and an enlarged eye-box. Research.

[147] Lin T., Zhan T., Zou J., Fan F., Wu S.T. (2020). Maxwellian near-eye display with an expanded eyebox. Opt. Express.

[148] Jo Y., Yoo C., Bang K., Lee B., Lee B. (2021). Eye-box extended retinal projection type near-eye display with multiple independent viewpoints. Appl. Opt..

[149] Xiong J., Li Y., Li K., Wu S.T. (2021). Aberration-free pupil steerable Maxwellian display for augmented reality with cholesteric liquid crystal holographic lenses. Opt. Lett..

[150] Ratnam K., Konrad R., Lanman D., Zannoli M. (2019). Retinal image quality in near-eye pupil-steered systems. Opt. Express.

[151] Jang C., Bang K., Li G., Lee B. (2018). Holographic near-eye display with expanded eye-box. ACM Trans. Graph. (TOG).

[152] Shi X., Liu J., Xiao J., Han J. (2021). Design of a compact waveguide eyeglass with high efficiency by joining freeform surfaces and volume holographic gratings. JOSA A.

[153] Weng Y., Zhang Y., Cui J., Liu A., Shen Z., Li X., Wang B. (2018). Liquid-crystal-based polarization volume grating applied for full-color waveguide displays. Opt. Lett..

[154] Lee Y.H., Tan G., Yin K., Zhan T., Wu S.T. (2018). Compact see-through near-eye display with depth adaption. J. Soc. Inf. Disp..

[155] Yoo C., Bang K., Chae M., Lee B. (2020). Extended-viewing-angle waveguide near-eye display with a polarization-dependent steering combiner. Opt. Lett..

[156] Ghasemi S., Otsuki M., Milgram P., Chellali R. (2017). Use of random dot patterns in achieving x-ray vision for near-field applications of stereoscopic video-based augmented reality displays. Presence.

[157] Gabbard J.L., Fitch G.M., Kim H. (2014). Behind the glass: Driver challenges and opportunities for AR automotive applications. Proc. IEEE.

[158] Kansaku K., Hata N., Takano K. (2010). My thoughts through a robot’s eyes: An augmented reality-brain–machine interface. Neurosci. Res..

[159] Kato H., Billinghurst M. Marker tracking and hmd calibration for a video-based augmented reality conferencing system. Proceedings of the Proceedings 2nd IEEE and ACM International Workshop on Augmented Reality (IWAR’99).

[160] Azuma R. (1993). Tracking requirements for augmented reality. Commun. ACM.

[161] Kato H., Billinghurst M., Poupyrev I., Imamoto K., Tachibana K. Virtual object manipulation on a table-top AR environment. Proceedings of the Proceedings IEEE and ACM International Symposium on Augmented Reality (ISAR 2000).

[162] Ke Y., Sukthankar R. PCA-SIFT: A more distinctive representation for local image descriptors. Proceedings of the 2004 IEEE Computer Society Conference on Computer Vision and Pattern Recognition (CVPR 2004).

[163] Kerawalla L., Luckin R., Seljeflot S., Woolard A. (2006). “Making it real”: Exploring the potential of augmented reality for teaching primary school science. Virtual Real..

[164] Kijima R., Ojika T. Transition between virtual environment and workstation environment with projective head mounted display. Proceedings of the IEEE 1997 Annual International Symposium on Virtual Reality.

[165] Koulieris G.A., Akşit K., Stengel M., Mantiuk R.K., Mania K., Richardt C. (2019). Near-eye display and tracking technologies for virtual and augmented reality. Computer Graphics Forum.

[166] Minoufekr M., Schug P., Zenker P., Plapper P.W. Modelling of CNC Machine Tools for Augmented Reality Assistance Applications using Microsoft Hololens. Proceedings of the ICINCO (2).

[167] Condino S., Montemurro N., Cattari N., D’Amato R., Thomale U., Ferrari V., Cutolo F. (2021). Evaluation of a Wearable AR Platform for Guiding Complex Craniotomies in Neurosurgery. Ann. Biomed. Eng..

[168] Milanović V., Kasturi A., Yang J., Hu F. (2017). A fast single-pixel laser imager for VR/AR headset tracking. Proceedings of the MOEMS and Miniaturized Systems XVI.

[169] Barz M., Kapp S., Kuhn J., Sonntag D. Automatic Recognition and Augmentation of Attended Objects in Real-time using Eye Tracking and a Head-mounted Display. Proceedings of the ACM Symposium on Eye Tracking Research and Applications.

[170] Evans G., Miller J., Pena M.I., MacAllister A., Winer E. (2017). Evaluating the Microsoft HoloLens through an augmented reality assembly application. Degraded Environments: Sensing, Processing, and Display.

[171] Fedosov A., Niforatos E., Elhart I., Schneider T., Anisimov D., Langheinrich M. Design and evaluation of a wearable AR system for sharing personalized content on ski resort maps. Proceedings of the 15th International Conference on Mobile and Ubiquitous Multimedia.

[172] Reuter C., Ludwig T., Mischur P. (2019). RescueGlass: Collaborative Applications involving Head-Mounted Displays for Red Cross Rescue Dog Units. Comput. Support. Coop. Work (CSCW).

[173] Medin R. (2018). Gesture-Driven Interaction in Head-Mounted Display AR: Guidelines for Design within the Context of the Order Picking Process in Logistic Warehouses. http://www.diva-portal.se/smash/get/diva2:1483645/FULLTEXT01.pdf.

[174] Tobias D. Data Visualisation using Augmented Reality with a Focus set on Head Mounted Displays and Collaborative Tasks. Proceedings of the International Symposium on NDT in Aerospace.

[175] Kim S., Suh Y., Lee Y., Woo W. (2006). Toward ubiquitous VR: When VR meets ubiComp. Proceedings of the 4th International Symposium on Ubiquitous VR.

[176] Andersson N., Argyrou A., Nägele F., Ubis F., Campos U.E., De Zarate M.O., Wilterdink R. (2016). AR-enhanced human-robot-interaction-methodologies, algorithms, tools. Procedia CIRP.

[177] Kiyokawa K., Takemura H., Yokoya N. A collaboration support technique by integrating a shared virtual reality and a shared augmented reality. Proceedings of the IEEE SMC’99 Conference Proceedings, 1999 IEEE International Conference on Systems, Man, and Cybernetics (Cat. No. 99CH37028).

[178] Kiyokawa K., Takemura H., Yokoya N. (2000). SeamlessDesign for 3D object creation. IEEE Multimed..

[179] Klein G., Drummond T. Sensor fusion and occlusion refinement for tablet-based AR. Proceedings of the Third IEEE and ACM International Symposium on Mixed and Augmented Reality.

[180] Klein G., Murray D.W. (2009). Simulating low-cost cameras for augmented reality compositing. IEEE Trans. Vis. Comput. Graph..

[181] Klein G., Murray D. Parallel tracking and mapping for small AR workspaces. Proceedings of the 2007 6th IEEE and ACM International Symposium on Mixed and Augmented Reality.

[182] Kiyokawa K., Kurata Y., Ohno H. (2001). An optical see-through display for mutual occlusion with a real-time stereovision system. Comput. Graph..

[183] Chan L.W., Kao H.S., Chen M.Y., Lee M.S., Hsu J., Hung Y.P. Touching the void: Direct-touch interaction for intangible displays. Proceedings of the SIGCHI Conference on Human Factors in Computing Systems.

[184] Jang S.W., Ko J., Lee H.J., Kim Y.S. (2018). A Study on Tracking and Augmentation in Mobile AR for e-Leisure. Mob. Inf. Syst..

[185] Fang W., Zheng L., Deng H., Zhang H. (2017). Real-time motion tracking for mobile augmented/virtual reality using adaptive visual-inertial fusion. Sensors.

[186] Irshad S., Awang D.R.B. A UX Oriented Evaluation approach for Mobile Augmented Reality Applications. Proceedings of the 16th International Conference on Advances in Mobile Computing and Multimedia.

[187] Loizeau Q., Danglade F., Ababsa F., Merienne F. (2021). Methodology for the Field Evaluation of the Impact of Augmented Reality Tools for Maintenance Workers in the Aeronautic Industry. Front. Virtual Real..

[188] Kraut R.E., Miller M.D., Siegel J. Collaboration in performance of physical tasks: Effects on outcomes and communication. Proceedings of the 1996 ACM Conference on Computer Supported Cooperative Work.

[189] Krum D.M., Suma E.A., Bolas M. (2012). Augmented reality using personal projection and retroreflection. Pers. Ubiquitous Comput..

[190] Kutter O., Aichert A., Bichlmeier C., Traub J., Heining S., Ockert B., Euler E., Navab N. (2008). Real-time volume rendering for high quality visualization in augmented reality. International Workshop on Augmented Environments for Medical Imaging Including Augmented Reality in Computer-Aided Surgery (AMI-ARCS 2008).

[191] Kolsch M., Bane R., Hollerer T., Turk M. (2006). Multimodal interaction with a wearable augmented reality system. IEEE Comput. Graph. Appl..

[192] Kuzuoka H. Spatial workspace collaboration: A SharedView video support system for remote collaboration capability. Proceedings of the SIGCHI Conference on Human Factors in Computing Systems.

[193] Lang P., Kusej A., Pinz A., Brasseur G. Inertial tracking for mobile augmented reality. Proceedings of the IMTC/2002. Proceedings of the 19th IEEE Instrumentation and Measurement Technology Conference (IEEE Cat. No. 00CH37276).

[194] LaViola J. (1999). Whole-Hand and Speech Input in Virtual Environments. CS-99-15. Master’s Thesis.

[195] Lee G.A., Kim G.J., Billinghurst M. (2007). Interaction design for tangible augmented reality applications. Emerging Technologies of Augmented Reality: Interfaces and Design.

[196] Lee G.A., Billinghurst M. A component based framework for mobile outdoor ar applications. Proceedings of the 12th ACM SIGGRAPH International Conference on Virtual-Reality Continuum and Its Applications in Industry.

[197] Lee G.A., Kim G.J., Park C.M. Modeling virtual object behavior within virtual environment. Proceedings of the ACM Symposium on Virtual Reality Software and Technology.

[198] Lee G.A., Billinghurst M., Kim G.J. Occlusion based interaction methods for tangible augmented reality environments. Proceedings of the 2004 ACM SIGGRAPH International Conference on Virtual Reality Continuum and Its Applications in Industry.

[199] Lee G.A., Nelles C., Billinghurst M., Kim G.J. Immersive authoring of tangible augmented reality applications. Proceedings of the Third IEEE and ACM International Symposium on Mixed and Augmented Reality.

[200] Santos M.E.C., Taketomi T., Yamamoto G., Rodrigo M., Mercedes T., Kato H. (2016). Augmented reality as multimedia: The case for situated vocabulary learning. Res. Pract. Technol. Enhanc. Learn..

[201] Lee G.A., Kim G.J., Billinghurst M. (2005). Immersive authoring: What you experience is what you get (wyxiwyg). Commun. ACM.

[202] Lee G.A., Yang U., Son W. (2006). Layered multiple displays for immersive and interactive digital contents. Proceedings of the International Conference on Entertainment Computing.

[203] Lee G.A., Kang H., Son W. Mirage: A touch screen based mixed reality interface for space planning applications. Proceedings of the 2008 IEEE Virtual Reality Conference.

[204] Lee G.A., Yang U., Son W., Kim Y., Jo D., Kim K.H., Choi J.S. (2010). Virtual reality content-based training for spray painting tasks in the shipbuilding industry. ETRI J..

[205] Lee G.A., Dünser A., Kim S., Billinghurst M. CityViewAR: A mobile outdoor AR application for city visualization. Proceedings of the 2012 IEEE International Symposium on Mixed and Augmented Reality-Arts, Media, and Humanities (ISMAR-AMH).

[206] Lee G.A., Dünser A., Nassani A., Billinghurst M. AntarcticAR: An outdoor AR experience of a virtual tour to Antarctica. Proceedings of the 2013 IEEE International Symposium on Mixed and Augmented Reality-Arts, Media, and Humanities (ISMAR-AMH).

[207] Lee J.Y., Rhee G.W., Seo D.W. (2010). Hand gesture-based tangible interactions for manipulating virtual objects in a mixed reality environment. Int. J. Adv. Manuf. Technol..

[208] Köse H., Güner-Yildiz N. (2021). Augmented reality (AR) as a learning material in special needs education. Educ. Inf. Technol..

[209] Oda O., Lister L.J., White S., Feiner S. Developing an augmented reality racing game. Proceedings of the 2nd International Conference on Intelligent Technologies for interactive enterTAINment.

[210] Schmalstieg D., Fuhrmann A., Hesina G., Szalavári Z., Encarnaçao L.M., Gervautz M., Purgathofer W. (2002). The studierstube augmented reality project. Presence Teleoperators Virtual Environ..

[211] Taehee Lee T., Handy A. Markerless inspection of augmented reality objects using fingertip tracking. Proceedings of the IEEE International Symposium on Wearable Computers.

[212] Lenhardt A., Ritter H. (2010). An augmented-reality based brain-computer interface for robot control. Proceedings of the International Conference on Neural Information Processing.

[213] Fiala M. (2004). Artag revision 1, a fiducial marker system using digital techniques. Natl. Res. Counc. Publ..

[214] Renner R.S., Velichkovsky B.M., Helmert J.R. (2013). The perception of egocentric distances in virtual environments-a review. ACM Comput. Surv. (CSUR).

[215] Creem-Regehr S.H., Stefanucci J.K., Thompson W.B., Nash N., McCardell M. Egocentric distance perception in the oculus rift (dk2). Proceedings of the ACM SIGGRAPH Symposium on Applied Perception.

[216] Kytö M., Mäkinen A., Tossavainen T., Oittinen P.T. (2014). Stereoscopic depth perception in video see-through augmented reality within action space. J. Electron. Imaging.

[217] Rosales C.S., Pointon G., Adams H., Stefanucci J., Creem-Regehr S., Thompson W.B., Bodenheimer B. Distance judgments to on-and off-ground objects in augmented reality. Proceedings of the 2019 IEEE Conference on Virtual Reality and 3D User Interfaces (VR).

[218] Rehman U., Cao S. (2016). Augmented-reality-based indoor navigation: A comparative analysis of handheld devices versus google glass. IEEE Trans. Hum.-Mach. Syst..

[219] Fischler M.A., Bolles R.C. (1981). Random sample consensus: A paradigm for model fitting with applications to image analysis and automated cartography. Commun. ACM.

[220] Favalora G.E. (2005). Volumetric 3D displays and application infrastructure. Computer.

[221] Feiner S., MacIntyre B., Haupt M., Solomon E. Windows on the world: 2D windows for 3D augmented reality. Proceedings of the 6th Annual ACM Symposium on User Interface Software and Technology.

[222] Feiner S., MacIntyre B., Seligmann D. (1993). Knowledge-based augmented reality. Commun. ACM.

[223] Feiner S., MacIntyre B., Höllerer T., Webster A. (1997). A touring machine: Prototyping 3D mobile augmented reality systems for exploring the urban environment. Pers. Technol..

[224] Fergason J.L. (1997). Optical System for a Head Mounted Display Using a Retro-Reflector and Method of Displaying an Image. U.S. Patent.

[225] Amin D., Govilkar S. (2015). Comparative study of augmented reality SDKs. Int. J. Comput. Sci. Appl..

[226] Fisher R.W. (1996). Head-Mounted Projection Display System Featuring Beam Splitter and Method of Making Same. U.S. Patent.

[227] Lepetit V., Fua P. (2006). Keypoint recognition using randomized trees. IEEE Trans. Pattern Anal. Mach. Intell..

[228] Leutenegger S., Chli M., Siegwart R.Y. BRISK: Binary robust invariant scalable keypoints. Proceedings of the 2011 International Conference on Computer Vision.

[229] Leventon M.E. (1997). A Registration, Tracking, and Visualization System for Image-Guided Surgery. Ph.D. Thesis.

[230] Kyza E.A., Georgiou Y. Digital tools for enriching informal inquiry-based mobile learning: The design of the TraceReaders location-based augmented reality learning platform. Proceedings of the 3rd Asia-Europe Symposium on Simulation & Serious Gaming.

[231] Carmigniani J., Furht B., Anisetti M., Ceravolo P., Damiani E., Ivkovic M. (2011). Augmented reality technologies, systems and applications. Multimed. Tools Appl..

[232] Lindeman R.W., Lee G., Beattie L., Gamper H., Pathinarupothi R., Akhilesh A. GeoBoids: A mobile AR application for exergaming. Proceedings of the 2012 IEEE International Symposium on Mixed and Augmented Reality-Arts, Media, and Humanities (ISMAR-AMH).

[233] Lingley A.R., Ali M., Liao Y., Mirjalili R., Klonner M., Sopanen M., Suihkonen S., Shen T., Otis B., Lipsanen H. (2011). A single-pixel wireless contact lens display. J. Micromechan. Microeng..

[234] Choi J., Kim Y., Lee M., Kim G.J., Nam Y., Kwon Y. k-MART: Authoring tool for mixed reality contents. Proceedings of the 2010 IEEE International Symposium on Mixed and Augmented Reality.

[235] Lindeman R.W., Sibert J.L., Hahn J.K. Towards usable VR: An empirical study of user interfaces for immersive virtual environments. Proceedings of the SIGCHI Conference on Human Factors in Computing Systems.

[236] Chong U., Alimardanov S. (2021). Audio augmented reality using unity for marine tourism. Proceedings of the International Conference on Intelligent Human Computer Interaction.

[237] Konopka B., Hönemann K., Brandt P., Wiesche M. (2022). WizARd: A No-Code Tool for Business Process Guidance through the Use of Augmented Reality. https://ceur-ws.org/Vol-3216/paper_258.pdf.

[238] Zhao S., Wang L., Song J. (2022). P-2.8: Research on multi-user interaction design in augmented reality. SID Symposium Digest of Technical Papers.

[239] Nebeling M., Speicher M. The trouble with augmented reality/virtual reality authoring tools. Proceedings of the 2018 IEEE International Symposium on Mixed and Augmented Reality Adjunct (ISMAR-Adjunct).

[240] Mladenov B., Damiani L., Giribone P., Revetria R. A short review of the SDKs and wearable devices to be used for ar application for industrial working environment. Proceedings of the World Congress on Engineering and Computer Science.

[241] Wells T., Houben S. Collabar–investigating the mediating role of mobile ar interfaces on co-located group collaboration. Proceedings of the 2020 CHI Conference on Human Factors in Computing Systems.

[242] Grandi J.G. Design of collaborative 3D user interfaces for virtual and augmented reality. Proceedings of the 2017 IEEE Virtual Reality (VR).

[243] Dong S., Behzadan A.H., Chen F., Kamat V.R. (2013). Collaborative visualization of engineering processes using tabletop augmented reality. Adv. Eng. Softw..

[244] Kim J.O., Kim J. (2018). Augmented Reality Tools for Integrative Science and Arts STEAM Education. Int. J. Pure Appl. Math..

[245] Kazanidis I., Tsinakos A., Lytridis C. (2017). Teaching mobile programming using augmented reality and collaborative game based learning. Proceedings of the Interactive Mobile Communication, Technologies and Learning.

[246] Chang Y.S., Hu K.J., Chiang C.W., Lugmayr A. (2020). Applying Mobile Augmented Reality (AR) to teach Interior Design students in layout plans: Evaluation of learning effectiveness based on the ARCS Model of learning motivation theory. Sensors.

[247] Sarkar P., Kadam K., Pillai J.S. Collaborative approaches to problem-solving on lines and angles using augmented reality. Proceedings of the 2019 IEEE Tenth International Conference on Technology for Education (T4E).

[248] Grandi J.G., Debarba H.G., Bemdt I., Nedel L., Maciel A. Design and assessment of a collaborative 3D interaction technique for handheld augmented reality. Proceedings of the 2018 IEEE Conference on Virtual Reality and 3D User Interfaces (VR).

[249] Akçayır M., Akçayır G., Pektaş H.M., Ocak M.A. (2016). Augmented reality in science laboratories: The effects of augmented reality on university students’ laboratory skills and attitudes toward science laboratories. Comput. Hum. Behav..

[250] Rekimoto J. Transvision: A hand-held augmented reality system for collaborative design. Proceedings of the International Conference on Virtual Systems and Multimedia.

[251] Oda O., Feiner S. Interference avoidance in multi-user hand-held augmented reality. Proceedings of the 2009 8th IEEE International Symposium on Mixed and Augmented Reality.

[252] Huynh D.N.T., Raveendran K., Xu Y., Spreen K., MacIntyre B. Art of defense: A collaborative handheld augmented reality board game. Proceedings of the 2009 ACM SIGGRAPH Symposium on Video Games.

[253] Nilsson S., Johansson B., Jonsson A. Using AR to support cross-organisational collaboration in dynamic tasks. Proceedings of the 2009 8th IEEE International Symposium on Mixed and Augmented Reality.

[254] Tseng P.Y., Haraldsson H., Belongie S. (2019). Annotate All! A Perspective Preserved Asynchronous Annotation System for Collaborative Augmented Reality. https://static1.squarespace.com/static/5c3f69e1cc8fedbc039ea739/t/5d0294ab169b78000195c0f1/1560450226075/32_Annotate_all__A_Perspective_Preserved_Asynchronous__Annotation_System_for_Collaborative_Augmented_Reality.pdf.

[255] Kasahara S., Heun V., Lee A.S., Ishii H. Second surface: Multi-user spatial collaboration system based on augmented reality. Proceedings of the SIGGRAPH Asia 2012 Emerging Technologies.

[256] Billinghurst M., Bowskill J., Morphett J. WearCom: A wearable communication space. Proceedings of the CVE.

[257] Stafford A., Piekarski W., Thomas B.H. Implementation of god-like interaction techniques for supporting collaboration between outdoor AR and indoor tabletop users. Proceedings of the 2006 IEEE/ACM International Symposium on Mixed and Augmented Reality.

[258] Gauglitz S., Nuernberger B., Turk M., Höllerer T. In touch with the remote world: Remote collaboration with augmented reality drawings and virtual navigation. Proceedings of the 20th ACM Symposium on Virtual Reality Software and Technology.

[259] Boonbrahm P., Kaewrat C., Boonbrahm S. (2020). Effective Collaborative Design of Large Virtual 3D Model using Multiple AR Markers. Procedia Manuf..

[260] Li X., Chen W., Wu Y. Distance-driven user interface for collaborative exhibit viewing in augmented reality museum. Proceedings of the The Adjunct Publication of the 32nd Annual ACM Symposium on User Interface Software and Technology.

[261] Poretski L., Lanir J., Arazy O. (2018). Normative tensions in shared augmented reality. Proc. ACM Hum.-Comput. Interact..

[262] Clergeaud D., Roo J.S., Hachet M., Guitton P. Towards seamless interaction between physical and virtual locations for asymmetric collaboration. Proceedings of the 23rd ACM Symposium on Virtual Reality Software and Technology.

[263] Oda O., Feiner S. 3D referencing techniques for physical objects in shared augmented reality. Proceedings of the 2012 IEEE International Symposium on Mixed and Augmented Reality (ISMAR).

[264] Mahmood T., Fulmer W., Mungoli N., Huang J., Lu A. Improving information sharing and collaborative analysis for remote geospatial visualization using mixed reality. Proceedings of the 2019 IEEE International Symposium on Mixed and Augmented Reality (ISMAR).

[265] Munoz-Cristóbal J.A., Gallego-Lema V., Arribas-Cubero H.F., Asensio-Pérez J.I., Martínez-Monés A. (2018). Game of Blazons: Helping teachers conduct learning situations that integrate web tools and multiple types of augmented reality. IEEE Trans. Learn. Technol..

[266] Muñoz-Cristóbal J.A., Prieto L.P., Asensio-Pérez J.I., Jorrín-Abellán I.M., Martínez-Monés A., Dimitriadis Y. (2013). GLUEPS-AR: A system for the orchestration of learning situations across spaces using augmented reality. Proceedings of the European Conference on Technology Enhanced Learning.

[267] bin Hanafi H.F., Said C.S., Ariffin A.H., Zainuddin N.A., Samsuddin K. (2016). Using a collaborative Mobile Augmented Reality learning application (CoMARLA) to improve Improve Student Learning. IOP Conference Series: Materials Science and Engineering.

[268] Dunleavy M., Dede C., Mitchell R. (2009). Affordances and limitations of immersive participatory augmented reality simulations for teaching and learning. J. Sci. Educ. Technol..

[269] Maimone A., Fuchs H. Encumbrance-free telepresence system with real-time 3D capture and display using commodity depth cameras. Proceedings of the 2011 10th IEEE International Symposium on Mixed and Augmented Reality.

[270] Gauglitz S., Nuernberger B., Turk M., Höllerer T. World-stabilized annotations and virtual scene navigation for remote collaboration. Proceedings of the 27th Annual ACM Symposium on User Interface Software and Technology.

[271] Guo A., Canberk I., Murphy H., Monroy-Hernández A., Vaish R. (2019). Blocks: Collaborative and persistent augmented reality experiences. Proc. ACM Interact. Mobile Wearable Ubiquitous Technol..

[272] Zhang W., Han B., Hui P., Gopalakrishnan V., Zavesky E., Qian F. CARS: Collaborative augmented reality for socialization. Proceedings of the 19th International Workshop on Mobile Computing Systems & Applications.

[273] Lien K.C., Nuernberger B., Höllerer T., Turk M. PPV: Pixel-point-volume segmentation for object referencing in collaborative augmented reality. Proceedings of the 2016 IEEE International Symposium on Mixed and Augmented Reality (ISMAR).

[274] Huang W., Billinghurst M., Alem L., Kim S. HandsInTouch: Sharing gestures in remote collaboration. Proceedings of the 30th Australian Conference on Computer-Human Interaction.

[275] Ou J., Fussell S.R., Chen X., Setlock L.D., Yang J. Gestural communication over video stream: Supporting multimodal interaction for remote collaborative physical tasks. Proceedings of the 5th International Conference on Multimodal Interfaces.

[276] Datcu D., Lukosch S.G., Lukosch H.K. Handheld augmented reality for distributed collaborative crime scene investigation. Proceedings of the 19th International Conference on Supporting Group Work.

[277] Tait M., Billinghurst M. (2015). The effect of view independence in a collaborative AR system. Comput. Support. Coop. Work (CSCW).

[278] Fang D., Xu H., Yang X., Bian M. (2020). An augmented reality-based method for remote collaborative real-time assistance: From a system perspective. Mob. Netw. Appl..

[279] Mora S., Boron A., Divitini M. (2012). CroMAR: Mobile augmented reality for supporting reflection on crowd management. Int. J. Mob. Hum. Comput. Interact. (IJMHCI).

[280] Adcock M., Feng D., Thomas B. Visualization of off-surface 3D viewpoint locations in spatial augmented reality. Proceedings of the 1st Symposium on Spatial User Interaction.

[281] Lincoln P., Welch G., Nashel A., Ilie A., State A., Fuchs H. Animatronic shader lamps avatars. Proceedings of the 2009 8th IEEE International Symposium on Mixed and Augmented Reality.

[282] Komiyama R., Miyaki T., Rekimoto J. JackIn space: Designing a seamless transition between first and third person view for effective telepresence collaborations. Proceedings of the 8th Augmented Human International Conference.

[283] Lehment N.H., Merget D., Rigoll G. Creating automatically aligned consensus realities for AR videoconferencing. Proceedings of the 2014 IEEE International Symposium on Mixed and Augmented Reality (ISMAR).

[284] Oda O., Elvezio C., Sukan M., Feiner S., Tversky B. Virtual replicas for remote assistance in virtual and augmented reality. Proceedings of the 28th Annual ACM Symposium on User Interface Software & Technology.

[285] Piumsomboon T., Lee G.A., Hart J.D., Ens B., Lindeman R.W., Thomas B.H., Billinghurst M. Mini-me: An adaptive avatar for mixed reality remote collaboration. Proceedings of the 2018 CHI Conference on Human Factors in Computing Systems.

[286] Piumsomboon T., Lee Y., Lee G., Billinghurst M. CoVAR: A collaborative virtual and augmented reality system for remote collaboration. Proceedings of the SIGGRAPH Asia 2017 Emerging Technologies.

[287] Teo T., Lee G.A., Billinghurst M., Adcock M. Hand gestures and visual annotation in live 360 panorama-based mixed reality remote collaboration. Proceedings of the 30th Australian Conference on Computer-Human Interaction.

[288] Thanyadit S., Punpongsanon P., Pong T.C. ObserVAR: Visualization system for observing virtual reality users using augmented reality. Proceedings of the 2019 IEEE International Symposium on Mixed and Augmented Reality (ISMAR).

[289] Sodhi R.S., Jones B.R., Forsyth D., Bailey B.P., Maciocci G. BeThere: 3D mobile collaboration with spatial input. Proceedings of the SIGCHI Conference on Human Factors in Computing Systems.

[290] Ong S., Shen Y. (2009). A mixed reality environment for collaborative product design and development. CIRP Ann..

[291] Irlitti A., Smith R.T., Von Itzstein S., Billinghurst M., Thomas B.H. Challenges for asynchronous collaboration in augmented reality. Proceedings of the 2016 IEEE International Symposium on Mixed and Augmented Reality (ISMAR-Adjunct).

[292] Azuma R., Baillot Y., Behringer R., Feiner S., Julier S., MacIntyre B. (2001). Recent advances in augmented reality. IEEE Comput. Graph. Appl..

[293] Baecker R.M. (1993). Readings in Groupware and Computer-Supported Cooperative Work: Assisting Human-Human Collaboration.

[294] Akussah M., Dehinbo J. (2018). Developing a Marker-based Handheld Augmented Reality Application for Learning Mathematics. EdMedia+ Innovate Learning.

[295] Roberto R., Lima J.P., Araújo T., Teichrieb V. Evaluation of motion tracking and depth sensing accuracy of the tango tablet. Proceedings of the 2016 IEEE International Symposium on Mixed and Augmented Reality (ISMAR-Adjunct).

[296] Maimone A., Fuchs H. Computational augmented reality eyeglasses. Proceedings of the 2013 IEEE International Symposium on Mixed and Augmented Reality (ISMAR).

[297] Mair E., Hager G.D., Burschka D., Suppa M., Hirzinger G. (2010). Adaptive and generic corner detection based on the accelerated segment test. Proceedings of the European Conference on Computer Vision.

[298] Mandeville J. A shared virtual environment for architectural design review. Proceedings of the CVE’96 Workshop Proceedings.

[299] Fernández-Caramés T.M., Fraga-Lamas P. (2018). Towards the Internet of smart clothing: A review on IoT wearables and garments for creating intelligent connected e-textiles. Electronics.

[300] Martínez H., Skournetou D., Hyppölä J., Laukkanen S., Heikkilä A. (2014). Drivers and bottlenecks in the adoption of augmented reality applications. J. Multimed. Theory Appl..

[301] Chessa M., Maiello G., Klein L.K., Paulun V.C., Solari F. Grasping objects in immersive Virtual Reality. Proceedings of the 2019 IEEE Conference on Virtual Reality and 3D User Interfaces (VR).

[302] Han D.T., Suhail M., Ragan E.D. (2018). Evaluating Remapped Physical Reach for Hand Interactions with Passive Haptics in Virtual Reality. IEEE Trans. Vis. Comput. Graph..

[303] Fotouhi J. (2020). Augmented Reality and Artificial Intelligence in Image-Guided and Robot-Assisted Interventions. Ph.D. Thesis.

[304] Wang Z., Bai X., Zhang S., Billinghurst M., He W., Wang Y., Han D., Chen G., Li J. (2021). The role of user-centered AR instruction in improving novice spatial cognition in a high-precision procedural task. Adv. Eng. Inform..

[305] Newman J., Wagner M., Bauer M., MacWilliams A., Pintaric T., Beyer D., Pustka D., Strasser F., Schmalstieg D., Klinker G. Ubiquitous tracking for augmented reality. Proceedings of the Third IEEE and ACM International Symposium on Mixed and Augmented Reality.

[306] Newman J., Schall G., Barakonyi I., Schürzinger A., Schmalstieg D. (2006). Wide-Area Tracking Tools for Augmented Reality. http://www.barakonyi.net/papers/ubisense_demo_pervasive06.pdf.

[307] Nilsen T., Looser J. Tankwar-Tabletop war gaming in augmented reality. Proceedings of the 2nd International Workshop on Pervasive Gaming Applications.

[308] Nilsson S., Johansson B. Acceptance of augmented reality instructions in a real work setting. Proceedings of the CHI’08 Extended Abstracts on Human Factors in Computing Systems.

[309] O’Conaill B. (1997). Characterizing, predicting and measuring video-mediated communication: A conversational approach. Video-Mediated Communication.

[310] Song E., Suaib N.M., Sihes A.J., Alwee R., Yunos Z.M. (2020). Design and development of learning mathematics game for primary school using handheld augmented reality. Iop Conf. Ser. Mater. Sci. Eng..

[311] Oh Y., Woo W. (2004). A unified application service model for ubihome by exploiting intelligent context-awareness. International Symposium on Ubiquitious Computing Systems.

[312] Oh Y., Shin C., Jung W., Woo W. (2005). The ubiTV application for a Family in ubiHome. Proceedings of the 2nd Ubiquitous Home Workshop.

[313] Ohshima T., Satoh K., Yamamoto H., Tamura H. (1998). Ar2 hockey: A case study of collaborative augmented reality. Proceedings of the VRAIS’98: Proceedings of the Virtual Reality Annual International Symposium.

[314] Olsson T., Lagerstam E., Kärkkäinen T., Väänänen-Vainio-Mattila K. (2013). Expected user experience of mobile augmented reality services: A user study in the context of shopping centres. Pers. Ubiquitous Comput..

[315] Ozuysal M., Calonder M., Lepetit V., Fua P. (2009). Fast keypoint recognition using random ferns. IEEE Trans. Pattern Anal. Mach. Intell..

[316] Pandey J., Liao Y.T., Lingley A., Mirjalili R., Parviz B., Otis B.P. (2010). A fully integrated RF-powered contact lens with a single element display. IEEE Trans. Biomed. Circuits Syst..

[317] Botha-Ravyse C., Lähtevänoja A., Luimula M. Collaborative AR application design for early childhood education. Proceedings of the EdMedia+ Innovate Learning. Association for the Advancement of Computing in Education (AACE).

[318] Parviz B.A. (2009). For your eye only. IEEE Spectr..

[319] Jana S., Molnar D., Moshchuk A., Dunn A., Livshits B., Wang H.J., Ofek E. Enabling Fine-Grained Permissions for Augmented Reality Applications with Recognizers. Proceedings of the 22nd USENIX Security Symposium (USENIX Security 13).

[320] Ruth K., Kohno T., Roesner F. Secure Multi-User Content Sharing for Augmented Reality Applications. Proceedings of the 28th USENIX Security Symposium (USENIX Security 19).

[321] Pierdicca R., Prist M., Monteriù A., Frontoni E., Ciarapica F., Bevilacqua M., Mazzuto G. (2020). Augmented reality smart glasses in the workplace: Safety and security in the fourth industrial revolution era. Proceedings of the International Conference on Augmented Reality, Virtual Reality and Computer Graphics.

[322] Ahn S., Gorlatova M., Naghizadeh P., Chiang M., Mittal P. Adaptive fog-based output security for augmented reality. Proceedings of the 2018 Morning Workshop on Virtual Reality and Augmented Reality Network.

[323] Chen S., Li Z., Dangelo F., Gao C., Fu X. A case study of security and privacy threats from augmented reality (ar). Proceedings of the 2018 International Conference on Computing, Networking and Communications (ICNC).

[324] Langfinger M., Schneider M., Stricker D., Schotten H.D. Addressing security challenges in industrial augmented reality systems. Proceedings of the 2017 IEEE 15th International Conference on Industrial Informatics (INDIN).

[325] Roesner F., Kohno T., Molnar D. (2014). Security and privacy for augmented reality systems. Commun. ACM.

[326] Wazir W., Khattak H.A., Almogren A., Khan M.A., Din I.U. (2020). Doodle-based authentication technique using augmented reality. IEEE Access.

[327] Lebeck K., Ruth K., Kohno T., Roesner F. Securing augmented reality output. Proceedings of the 2017 IEEE Symposium on Security and Privacy (SP).

[328] Zhang X., Slavin R., Wang X., Niu J. Privacy assurance for android augmented reality apps. Proceedings of the 2019 IEEE 24th Pacific Rim International Symposium on Dependable Computing (PRDC).

[329] McPherson R., Jana S., Shmatikov V. No escape from reality: Security and privacy of augmented reality browsers. Proceedings of the 24th International Conference on World Wide Web.

[330] Roesner F., Kohno T. Security and Privacy for Augmented Reality: Our 10-Year Retrospective. Proceedings of the VR4Sec: 1st International Workshop on Security for XR and XR for Security.

[331] Lebeck K., Ruth K., Kohno T., Roesner F. Towards security and privacy for multi-user augmented reality: Foundations with end users. Proceedings of the 2018 IEEE Symposium on Security and Privacy (SP).

[332] Dissanayake V.D. (2019). A Review of Cyber Security Risks in an Augmented Reality World.

[333] Lebeck K. (2019). Security and Privacy for Emerging Augmented Reality Technologies. Ph.D. Thesis.

[334] Marques B., Silva S., Alves J., Rocha A., Dias P., Santos B.S. (2022). Remote collaboration in maintenance contexts using augmented reality: Insights from a participatory process. Int. J. Interact. Des. Manuf..

[335] Kang J.Y.M., Kim J.E., Lee J.Y., Lin S.H. (2022). How mobile augmented reality digitally transforms the retail sector: Examining trust in augmented reality apps and online/offline store patronage intention. J. Fash. Mark. Manag. Int. J..

[336] Alimamy S., Gnoth J. (2022). I want it my way! The effect of perceptions of personalization through augmented reality and online shopping on customer intentions to co-create value. Comput. Hum. Behav..

[337] Ghafoori M., Shadnoosh N., Karamati M.A. (2022). The effect of co-creation in the face of augmented reality on perceived risk, perceived trust. J. Invest. Knowl..

[338] Butt G.Q., Sayed T.A., Riaz R., Rizvi S.S., Paul A. (2022). Secure Healthcare Record Sharing Mechanism with Blockchain. Appl. Sci..

[339] Djenouri Y., Belhadi A., Srivastava G., Lin J.C.W. (2021). Secure collaborative augmented reality framework for biomedical informatics. IEEE J. Biomed. Health Inform..

[340] Lukosch S., Lukosch H., Datcu D., Cidota M. On the spot information in augmented reality for teams in the security domain. Proceedings of the 33rd Annual ACM Conference Extended Abstracts on Human Factors in Computing Systems.

[341] Althewaynee H.B., Hamood M.M., Hussein H.A. (2022). A systematic review of using augmented reality in tourism between 2017 and 2021. Res. J. Anal. Invent..

[342] Ali T., Alam M., Nauman M., Ali T., Ali M., Anwar S. (2011). A scalable and privacy preserving remote attestation mechanism. Inf.-Int. Interdiscip. J..

[343] Syed T.A., Jan S., Musa S., Ali J. Providing efficient, scalable and privacy preserved verification mechanism in remote attestation. Proceedings of the 2016 International Conference on Information and Communication Technology (ICICTM).

[344] Siddiqui M.S., Syed T.A., Nadeem A., Nawaz W., Alkhodre A. (2022). Virtual Tourism and Digital Heritage: An Analysis of VR/AR Technologies and Applications. Int. J. Adv. Comput. Sci. Appl..

[345] Tola E., Lepetit V., Fua P. (2009). Daisy: An efficient dense descriptor applied to wide-baseline stereo. IEEE Trans. Pattern Anal. Mach. Intell..

[346] Träskbäack M., Haller M. Mixed reality training application for an oil refinery: User requirements. Proceedings of the 2004 ACM SIGGRAPH International Conference on Virtual Reality Continuum and Its Applications in Industry.

